# Composite materials with recycled fibers: Evaluation of the effect of different fibers on gypsum composites

**DOI:** 10.1038/s41598-026-46588-6

**Published:** 2026-04-13

**Authors:** Mark Hemphill, Alexander L. Yarin

**Affiliations:** 1 United States Gypsum, 700 North US Highway 45, Libertyville, IL 60048-1296 USA; 2 Department of Mechanical and Industrial Engineering, University of Illinois at Chicago, 842 W. Taylor St, Chicago, IL 60607-7022 USA

**Keywords:** Gypsum, Paper, Hemp, Fiberglass, Fiber, Fracture toughness, Engineering, Materials science

## Abstract

The use of alternate more sustainable materials in building construction products has been a focus for the last two decades. Defining new methods to utilize materials destined for landfill while not increasing cost and discovering beneficial reuse for such materials is commended in today’s industry. This work explores three types of fibers (paper, hemp and fiberglass) with the goal of improving overall performance and sustainability of wallboard by means of making gypsum-fiber composites. Circular composite disks containing 0–2 wt% fibers in gypsum were tested with a biaxial bending apparatus. Fracture toughness was also calculated in the case of 1.5 wt% fiber loading. When recycled paper fibers were utilized in composites, the resulting composite exhibited a shift towards a less brittle material. The improvement in load resistance and deflections in the biaxial bending test was comparable to those of gypsum composites with high-energy manufactured fiberglass. Within the range investigated, hemp fibers, while sustainable, did not provide improved performance and may need to be refined further, which would add more cost. Fracture toughness improved with all fiber loadings with fiberglass revealing the highest toughness. SEM images suggest that the paper fibers had good surface bonding with the gypsum matrix, while the fiberglass appeared less, indicating different mechanisms to achieve improved performance.

**Supplementary Information:**

The online version contains supplementary material available at 10.1038/s41598-026-46588-6.

## Introduction

Gypsum is a widely abundant material in the Earth’s crust. Gypsum contains ~ 20 wt% of water as per Geraldo et al.^1^, i.e., is hydrated according to the following reaction:

1 $$CaS{O_4}\left( {Calcium{\text{ }}Sulphate} \right){\text{ }} + {\text{ }}2{\text{ }}{H_2}O{\text{ }}\left( {liquid} \right)\rightarrow\left( {heat} \right)\;CaS{O_4}\cdot 2{\text{ }}{H_2}O{\text{ }}\left( {gypsum} \right)$$

This reaction proceeds in the 125–180 °C range, is endothermic and is known as calcination. Calcined gypsum is commonly referred to as stucco or “Plaster of Paris” which is a hygroscopic material. The reaction is reversible, implying water removal from stucco in a reverse reaction counterpart of reaction (1). The reverse reaction, accordingly, is exothermic, i.e., is accompanied by heat release. The reverse reaction is widely used in the industry to manufacture wallboard.

Wallboard is a common construction material in building residential homes as well as in commercial construction. It is also known as a brittle material, which generally means that being loaded, it could suddenly break at a relatively small deformation. Accordingly, when the wallboard is dropped or attached with screws, the gypsum matrix will usually break due to a brittle fracture failure. Typically, Young’s modulus of wallboard is of the order of 1.75–2.5 GPa (Cramer et al.^2^.

Wallboard is made by feeding stucco with water and other functional raw materials into a mixer and depositing the resulting highly mixed slurry onto a table (a moving belt), where the slurry layer is sandwiched by two sheets of wallboard paper pulled by conveyor belts. Gypsum slurries, as described by Sinha Ray et al.^3^ and Dannessa et al.^4^, are non-Newtonian shear-thinning (pseudoplastic) fluids. Generally, when wallboard is made, there is a denser layer that is deposited on the face paper to make harder edges to improve the installation of the final brittle product. Typically, foam is injected into the edges to make the edges more acceptable to the screws that are used to install the wallboard. Overall, there is a need to design such composite material to improve durability and strength of the wallboard edges.

The use of fibers in wallboard has been known for several decades. Dalmay et al.^5^ studied the influence of hemp and flax fibers on the properties of hydrated gypsum. Their conclusions revealed that the fibers affected the set properties of hydrating gypsum matrix by retarding the exothermic reaction when the fibers were untreated. Such fibers also changed the matrix behavior from a brittle one to a material with a much better resistance to cracking. Al-Rifaie et al.^6^ utilized palm fibers in a gypsum cast. They concluded that the palm fibers improved the strength to rupture and the overall toughness of the composite material. In addition, Abir et al.^7^ employed jute fibers embedded in a gypsum matrix, which improved the strength at rupture as well as the tensile properties and transformed the composite into a less brittle material. It should be emphasized that hemp, jute, and flax fibers are hygroscopic, as reported by Bezerra De Souza et al.^8^. The previous works with different fibers in a gypsum matrix are summarized in Table 1.

**Table 1:**
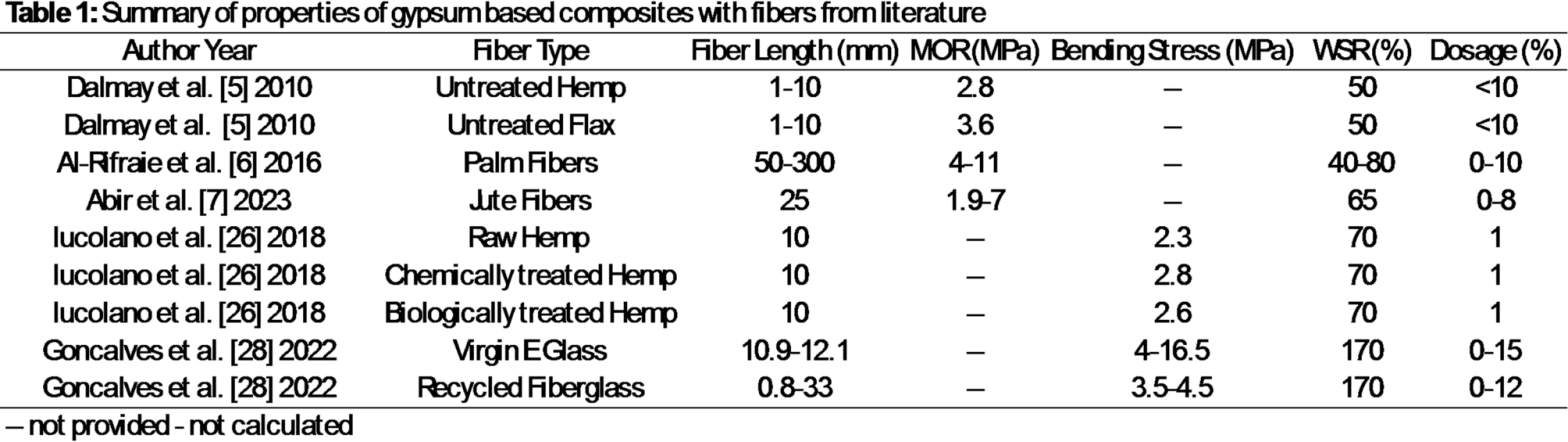
.

All the above-mentioned gypsum composites required either pre-treatment of the fibers to improve performance and/or the addition of the fibers at a cost of mixing the materials together. Also, it is known that in the process of adding fibers into a gypsum material, the fibers could imbibe a significant amount of water and thus, change the slurry flowability, a very important parameter in wallboard manufacturing. The higher water loading required to manufacture wallboard makes the process less sustainable and results in higher costs as well. Therefore, injecting a necessary amount of fibers into the matrix would, essentially, require a novel process. A slurry of cellulose fibers is a non-Newtonian fluid and thus, mixing two non-Newtonian slurries would typically require secondary mixing to attain good fiber distribution throughout the matrix material. As Ventura et al.^9^ suggested, when cellulose pulp is pumped in turbulent regime, the pulp-slurry mixture behaves more like a Newtonian fluid. Mixing water, a Newtonian fluid, with gypsum slurry is done routinely in wallboard manufacturing. An increase of the flow velocity of cellulose pulp to reach transition to turbulence before encountering the gypsum slurry, potentially should allow an easier mixing and a better fiber distribution without energy-ineffective secondary mixing. The additional positive outcomes of such an approach are in the use of recycled fibers, which not only help to enhance the strength of the final product (cf. the review of Haba et al.^10^, but also provide benefits of reuse of the recycled fibers that are collected by millions of homeowners weekly.

The present work elucidates how an addition of fibers into a gypsum matrix improves the gypsum composite properties making it less brittle and improves its fracture toughness. The work also offers a modified biaxial disk-bending test to determine the composite properties. Unlike previous research into fiber reinforced gypsum, the focus here is on innovative wallboard-based formulations compared to those utilized in modern-day wallboard manufacturing. Utilizing any fiber beyond 3–5% is not practical in modern wallboard manufacturing environment. At these dosages, the wallboard process would significantly have to be changed to manufacture drywall. The present work identifies specific dosage ranges that are practical and provides data that suggest a mechanical response transition from brittle behavior to less brittle responses that may correlate lesser drywall damage which is important to end users and is reasonable from a cost position.

## Experimental

### Materials

Gypsum used for all the experiments originated from a synthetic gypsum source of high quality. Synthetic gypsum is made from desulfurization of stacks from power plants that burn coal Koralegedara et al.^11^. The gypsum purity of the synthetic gypsum used during the bench experiments in the present work was 97.1 wt%.

Cellulose (paper) fibers used to prepare pulps originated from recycled wallboard paper, as per Fig. S1 (left) in Supplementary Information (SI). Wallboard paper is usually made from 100 wt% recycled fibers collected from urban recycling programs, where curbside pickup of household waste, such as Amazon packaging, are collected and sorted and resold back to paper mills to manufacture wallboard paper. It should be emphasized that with an increase in online shopping with companies like Amazon and a sharpened focus on sustainability, there is an need to utilize packaging waste that is generated from these businesses. When reviewing Amazons’ 2022 sustainability report, one can observe that Amazon reduced by 60,000 tons cardboard usage which accounts for 7–10 wt% reduction by means of their innovative PackOpt tool in North America (Amazon^12^. Using the lower 7 wt% value, that amounts to Amazon using approximately 850,000 tons of cardboard to ship their packages in North America. Any means that lead to the use of this recyclable material in a beneficial manner would be welcomed, especially if the usage improves performance of manufactured material, as in the present case of wallboard.

The hemp hurd fines cellulose fibers (2 mm and smaller in one-pound bags) were purchased online from Bulk Hemp Warehouse. These fibers are shown in Fig. S1 (center) in SI.

The fiberglass that was used in the study was 12.7 mm (½ inch) fiberglass Duracore 300 from Johns Mansville Company. Fiberglass is routinely used in fire-rated products in gypsum wallboard manufacturing. The material is presented in Fig. S1 (right) in SI. These fibers are bundled together and are 12.7 mm (½ inch) in length and ~ 16 μm in diameter according to the online information. This type of fiberglass is made from E glass.

The polymethyl methacrylate (PMMA) used in this study was a cast PMMA acrylic shown in Fig. S2 in SI, which was purchased from McMaster Carr catalog.

#### Mixing and disk forming

Gypsum is a brittle material and when subject to bending it fails similarly to other brittle materials, e.g., ceramics (Garcıa-Prieto et al.^13^). Brittle failure is explained in the framework of the Griffith theory (Cherepanov^14^, Barenblatt^15^, Yarin et al.^16^). Paper fibers would improve gypsum strength only if they are uniformly distributed throughout the gypsum matrix resulting in a homogeneous composite material. This was demonstrated by Hernandez-Olovares et al.^17^ where 1–3 wt% of sisal fibers improved the overall flexural strengths and fracture toughness of composite material.

In the present work, gypsum, fibers and other raw materials for set control were mixed at a water-to-stucco ratio (WSR) of ~ 110 wt%. The water used in the experiments was tap water. A high-shear mixer used was a Waring blender depicted in Fig. S3 (left) in SI. The liquid components were loaded into the mixer at ~ 20,000 rpm (equivalent to 333.33 rev/s and thus, the shear rate of $\mathop \gamma \limits^{\cdot } =333.33 \times 2\pi =2093\;{s^{ - 1}}$), while the powder was dumped into the mixer and was allowed to soak ~ 10 s. Then, the mixer was turned on to mix the slurry for ~ 10 s. After that, the slurry was quickly and carefully poured into plastic and steel forms, and any excess slurry was screeded off to make a smooth surface for the cast gypsum disks and beams. The dimensions of the forms were 102 mm (4 inch) ID and 8.8 mm (~ 0.345 inch) thickness (Fig. S3, center) in SI and 25.4 mm $\times$ 25.4 mm $\times$ 210 mm (1 inch $\times$ 1 inch $\times$ 8.25 inch) (Fig. S3, right) in SI.

Once the specimens were cast, they were removed from the forms within 3–5 min. The disks and beams were left to hydrate for a minimum of 20 min before being transferred into a 110 ºF oven for a minimum of 2 days to ensure total drying. The specimens were put into a 75 ºF/50 wt% humidity room for a minimum of 16 h before testing. It should be emphasized that the drying procedure followed a standard protocol for gypsum testing in the ASTM 1396 standard. Specimen dosages are listed in Table 2, whereas Table 3 lists the characteristics of the paper fibers, fiberglass and hemp fibers used in the cast.

It should be emphasized that water absorption is a feature of gypsum that is often measured in studies incorporating properties into gypsum. The present work is focused on introducing a new attribute of durability to gypsum matrix, which is characterized via test results in fracture toughness as well as the disk biaxial bending. While this study is focused on durability properties, the effects on water absorption due to fiber addition into gypsum slurry are outside the present scope and are recommended for future work.

**Table 2:** Specimen loadings.

Name	Mix proportions/(g)			
	Gypsum	Water	WSR/(wt%)	Fibers/(wt%)
Control	400	447	112	0
Paper fibers	400	447	112	0.5
	400	447	112	1
	400	447	112	1.5
	400	447	112	2
Hemp fibers	400	447	112	0.5
	400	447	112	1
	400	447	112	1.5
	400	447	112	2
Fiberglass	400	447	112	0.5
	400	447	112	1
	400	447	112	1.5
	400	447	112	2

### Disk and beam testing procedures

The disks and beams were tested using a Universal Testing Machine (UTM) with a point loading applied at the center of the disk, as depicted in Fig. S4 (left) in SI. The disks were 102 mm (4 inch) in diameter and were placed over an unsupported 76 mm (3 inch) diameter hole, as depicted in Fig. S4 (center). To calculate Young’s modulus E, all beams were subjected to 3-point or 4-point bending at the center of a 254 mm (10 inch) unsupported span. Fracture toughness beams were subject to 3-point bending at the center over an 203 mm (8 inch) unsupported span. The notch that was used was 6.4 mm (¼ inch) high and 2.54 mm (0.1 inch) wide. The tip of the notch was machined to a point as shown in Fig. S5. The UTM speed was 1 inch/min during all tests. The conditions in the room were controlled at 75 ºF and 50 wt% Relative Humidity.

The deflection was measured by positioning a digital dial indicator, CDICOR3110, which has a travel distance of 25.4 mm (1 inch) and a resolution of 0.001 mm. The dial indicator was placed under the disk center and beam center, as shown in Fig. S4 (left and right) in SI.

**Table 3:** Fiber characteristics.

Fiber	Count, millions/g of pulp	Mean weighted length (µm)	Mean fiber width (µm)	Fines, millions/g of pulp
Paper fibers	5.8	1274.0	23.9	366.6
Hemp fibers	0.3	352.7	39.8	67.8
Fiberglass	--	500	16	--

#### Membrane bending test: Theoretical

Construction with wallboard often results in damage that leads to waste that cost contractors and homeowners time and money. Wallboard is a brittle material that is made predominately from gypsum and undergoes significant bending and shear forces when being carried, delivered and installed on a job site. A biaxial bending testing method was designed which uses a point load at the center of an unsupported disk to quantify the biaxial load resistance and deflection of gypsum casts with fiber reinforcements.

Historically, biaxial bending was performed on metals and such minerals as Leucite used in dental ceramics. Miura et al.^18^ compared 3-point, 4-point and biaxial bending in their research work on dental ceramics. Their conclusions revealed that biaxial testing was not significantly different than 3-point and 4-point bending when considering the Weibull coefficients of shape and scale. Gypsum is typically tested for break strengths using 3-point and 4-point bending and the present work’s uniqueness is in utilizing biaxial bending to determine and optimize load resistance as it relates to fiber reinforcement of gypsum matrices. This type of test will provide qualitative insights into the real handling of wallboards which is rarely uniaxial because they are often being exposed to multi-axial bending loads from manufacturing plant to the final-use case when they are installed onto a wall.

The composite material is implied to be homogeneous, whereas the deflections are small and axisymmetric. Flexural Rigidity D of a membrane (a disk, in the present case) is defined as

3.1 $${\mathrm{D=}}\frac{{{\mathrm{E}}{{\mathrm{h}}^{\mathrm{3}}}}}{{{\mathrm{12(1-}}{{{\boldsymbol{\upnu}}}^{\mathrm{2}}}{\mathrm{)}}}}$$

Most equations did not convert symbols nu and pi.  Equation 3.1 is not the same as manuscript.  Needs to be fixed per manuscript. The denominator has error..  12(1-nu^2)  where Young’s modulus is denoted by E, h is the disk thickness, and Poisson’s ratio by ν (Landau and Lifshitz^18^ and Yarin et al.^16^).

In the present tests a pointwise load

3.2 $${\mathrm{F}} = - {{\text P}}\frac{{{\boldsymbol{\delta }}\left( {\mathrm{r}} \right)}}{{{{2\pi r}}}}$$

is applied to the center of the disk which is unsupported, except at the circumference.

In Eq. (3.2) δ(r) is the Dirac delta function, r is the polar coordinate reckoned for the disk center, and *P* > 0 is the force magnitude, and the force is directed downward, which is expresses by the minus sign. In this case, the general membrane equation reads (Landau and Lifshitz^18^ and Yarin et al.^16^

3.3 $$D{\nabla ^4}w - \nabla \cdot \left[ {\mathrm{h}\boldsymbol{\upsigma}\cdot \nabla w} \right]=F$$

where **σ** is the stress tensor in the membrane.

The first term on the left in Eq. (3.3) is caused by the moment of elastic forces in the membrane cross-section, which resists bending. On the other hand, the second term in Eq. (3.3) is caused by the resistance of the elastic force associated with the membrane stretching. It should be emphasized that the first term in Eq. (3.3) is linear with the local bending amplitude w, whereas the second term in Eq. (3.3) is nonlinear.

When the bending amplitude w is relatively small, the nonlinear term in Eq. (3.3) can be neglected and accounting for Eq. (3.2) it takes the form

3.4 $$\frac{{\mathrm{1}}}{{\mathrm{r}}}\frac{{\mathrm{d}}}{{{\mathrm{dr}}}}\left\{ {{\mathrm{r}}\frac{{\mathrm{d}}}{{{\mathrm{dr}}}}\left[ {\frac{{\mathrm{1}}}{{\mathrm{r}}}\frac{{\mathrm{d}}}{{{\mathrm{dr}}}}\left( {{\mathrm{r}}\frac{{{\mathrm{dw}}}}{{{\mathrm{dr}}}}} \right)} \right]} \right\}{\mathrm{=}}-\frac{{\mathrm{P}}}{{\mathrm{D}}}\frac{{{{\boldsymbol{\updelta}}}\left( {\mathrm{r}} \right)}}{{{{2\boldsymbol{\uppi}\text r}}}}$$

The boundary conditions imposed of the solution of Eq. (3.4) imply that the central deflection is finite and its amplitude is maximal, the circumference at r = a is supported, and hinged, i.e., according to (Landau and Lifshitz^18^

3.5 $${\mathrm{at}}\;{\mathrm{r=0:}}\;\left| {\mathrm{w}} \right|{\mathrm{<}}\infty {\mathrm{;}}\quad {\mathrm{at}}\;{\mathrm{r=0:}}\;\;\frac{{{\mathrm{dw}}}}{{{\mathrm{dr}}}}{\mathrm{=0;}}\quad {\mathrm{at}}\;{\mathrm{r=a:}}\quad {\mathrm{w=0;}}\quad {\mathrm{at}}\;{\mathrm{r=a:}}\quad \frac{{{{\mathrm{d}}^{\mathrm{2}}}{\mathrm{w}}}}{{{\mathrm{d}}{{\mathrm{r}}^{\mathrm{2}}}}}{\mathrm{+}}\frac{{{\boldsymbol{\upnu}}}}{{\mathrm{r}}}\frac{{{\mathrm{dw}}}}{{{\mathrm{dr}}}}{\mathrm{=0}}$$

Solution of the problem (3.4) and (3.5) yields the following expression for the central deflection w_0_ at *r* = 0

3.6 $${{\mathrm{w}}_{\mathrm{0}}}{\mathrm{=-}}\frac{{{\mathrm{P}}{{\mathrm{a}}^{\mathrm{2}}}}}{{{{16\boldsymbol{\uppi}D}}}}\frac{{{{(3+\boldsymbol{\upnu})}}}}{{{{(1+\boldsymbol{\upnu})}}}}$$

Again, nu and pi did not convert Substituting Eq. (3.1) into Eq. (3.6), one finds the following expression for E

3.7 $${\mathrm{E=}}\frac{{{\mathrm{3P}}{{\mathrm{a}}^{\mathrm{2}}}}}{{{{4\boldsymbol{\uppi}}}\left| {{{\mathrm{w}}_{\mathrm{0}}}} \right|{{\mathrm{h}}^{\mathrm{3}}}}}\left( {{{3+\boldsymbol{\upnu}}}} \right)\left( {{{1-\boldsymbol{\upnu}}}} \right)$$

Again nu and pi did not convert.  In the limit of relatively stiff specimens Eq. (3.7) can be employed to find E by measuring the central deflection magnitude $\left| {{w_0}} \right|$by using the Universal testing machine in the linear elasticity regime. This provides an alternate method to determine Young’s modulus in addition to the traditional 3- and 4-point bending tests.

In the limit of dominating nonlinear effect, the first term in Eq. (3.3) can be neglected because stretching causes the dominant resistance to bending. Accordingly, in the axisymmetric case Eq. (3.3) reduces to

3.8 $$\frac{1}{r}\frac{d}{{dr}}\left( {h{\sigma _{\tau \tau }}r\frac{{dw}}{{dr}}} \right)+F=0$$

where σ_ττ_ is the normal stress component directed along the membrane generatrix, with the local unit vector **τ**.

It should be emphasized that the stress σ_ττ_ is caused by the nonlinear effect - stretching of the membrane generatrix in comparison to its initial straight unstretched configuration. Accordingly, the membrane strain is found as ${\varepsilon _{\tau \tau }}=\sqrt {1+{{\left( {dw/dr} \right)}^2}} - 1 \approx \left( {1/2} \right){\left( {dw/dr} \right)^2}$, which yields the stress ${\sigma _{\tau \tau }}=\left( {E/2} \right){\left( {dw/dr} \right)^2}$. Then, using Eq. (3.2), Eq. (3.8) takes the following form:

3.9 $$\frac{1}{r}\frac{d}{{dr}}\left[ {hr\frac{E}{2}{{\left( {\frac{{dw}}{{dr}}} \right)}^2}\frac{{dw}}{{dr}}} \right] - P\frac{{\delta \left( r \right)}}{{2\pi r}}=0$$

This equation is integrated using the boundary condition

3.10 $$\quad {\mathrm{at}}\;{\mathrm{r=a:}}\quad {\mathrm{w=0}}$$

and considering that, if *P* = 0, the membrane profile degenerates to $w \equiv 0$.

Solution of Eq. (3.9) subjected to the boundary condition (3.10) yields the following expression for the central deflection w_0_ at *r* = 0

3.11 $${w_0}= - \frac{3}{2}{\left( {\frac{P}{{\pi Eh}}} \right)^{1/3}}{a^{2/3}}$$

which yields the following expression for E

3.12 $$E=\frac{9}{4}\frac{{P{a^2}}}{{\pi h{{\left| {{w_0}} \right|}^3}}}$$

This expression for E corresponding to the limit of the nonlinear stretching resistance to bending is worth of comparing with the one corresponding to the linear resistance given by Eq. (3.7), say, with ν = 1/2, i.e.,

3.13 $${\mathrm{E=}}\frac{{{\mathrm{21P}}{{\mathrm{a}}^{\mathrm{2}}}}}{{{{16\boldsymbol{\uppi}}}\left| {{{\mathrm{w}}_{\mathrm{0}}}} \right|{{\mathrm{h}}^{\mathrm{3}}}}}$$

Again, pi did not convert. The comparison reveals that the values of E predicted by Eq. (3.12) in the nonlinear regime when $\left| {{w_0}} \right|\sim h$cannot differ much from the one predicted by the linear theory resulting in Eq. (3.13).

## Results and discussion

### Young’s modulus of pure gypsum casts

Gypsum is a brittle material with E in the 1–4 GPa range depending on the amount of water used to make the cast. Generally, more water in the cast will make a weaker cast gypsum due to the most-water voids which lead to lower stiffness of the material. In the 3- and 4-point bending tests with beams, gypsum casts with the WSR ~ 110 wt% were employed. The corresponding results for E are compared below with the results of the membrane bending test described in the previous sections when a value of ν = 0.2 was used as per Dalmay^5^, whereas for PMMA disk-like membranes ν = 0.35, Dowling^20^. Table 4 summarizes the results of the 3- and 4-point bending tests with beams and of the membrane bending with the disks. It should be emphasized that disks of different diameters were used to verify that the measured value of E does not depend on the disk diameter. This trend is shown in Table 4 and is comprised of data for multiple samples of gypsum and PMMA. The 3-point tests were conducted with ten gypsum beams and the 4-point tests- with twelve gypsum beams. For gypsum disks, there were six 79 mm (3.1 inch) disks and eight 102 mm (4 inch) disks; for PMMA- six beams and five disks were used.

**Table 4:** Young’s modulus (E) of pure gypsum casts and PMMA.

Material	Beam (GPa)			Disk (GPa)	
	3- Point	4-Point		79 mm disk	102 mm disk
Gypsum	2.95$\pm$0.153		3.01$\pm$0.065	1.42$\pm$0.214	1.41$\pm$0.205
PMMA			3.01$\pm$0.201		2.91$\pm$0.106

The results reveal that the Young’s modulus value determined in the membrane experiments with gypsum disks is about one half of that measured in the 3- and 4-point bending tests. It is known that 3-point test introduces shear into the specimen when under load. The 4-point bending test eliminates the effect of shear forces, and reveals that their effect was minor, since the E values are so close. A significant difference in the values of E determined by the 3- and 4-point bending tests and the membrane test is hypothesized to arise due to the microscopic water voids that are in the material. Under the biaxial bending conditions in the membrane test, such voids lower the stiffness of the material and with biaxial stresses applied lead to premature failure. To test this hypothesis, PMMA materials were prepared in the same shape and form as the gypsum casts, namely beams and disks as shown in Fig. S6 in SI. PMMA possesses a similar Young’s modulus as gypsum, however, is much more dense material with much lower porosity. These PMMA specimens were used in the 4-point bending test and the membrane test.

According to Dowling^20^, PMMA possesses Young’s modulus of E = 2.3–3.2 GPa and Poisson’s ratio of ν = 0.35. Both results for PMMA presented in Table 4 agree well with this range. The membrane test with disks revealed a lesser value of E than the 4-point bending test, albeit within ~ 3%. PMMA is a brittle isotropic material, which has much less voids than gypsum. Accordingly, in the biaxial bending in the membrane test, PMMA behaves similarly to the 4-point bending test. On the other hand, the difference between the two materials (PMMA and gypsum) is quite significant.

To interpret the differences in the membrane test results, it is required to understand the differences in behavior of the two homogenous materials. PMMA is well documented and provides an example of good brittle material to determine its Young’s modulus and compared to that of the neat gypsum matrix, which is useful in comparing the two geometries. The water voids content and thus the porosity in gypsum matrix is much higher than that of PMMA. Aljubouri et al.^21^ provided the porosity values for three different gypsums. Accordingly, the technical plasters which had a WSR between 45% and 50%, possess a porosity of ~ 38%. To contrast that, Figuerôa et al.^22^ tested PMMA using two distinct methods to polymerize the material, and both methods yielded porosities ~ 1.5% which is much lower than that of a gypsum cast at WSR of 45%–50%. A higher WSR would be expected to increase water voids in the gypsum cast. This highlights the distinct context for the differences between the behavior of the two materials under biaxial loading.

Comparing the two materials using the beam test with the 3-point and 4-point bending revealed good correlation. However, when the disks under biaxial bending were compared, the value of E was off significantly. When the disks were broken, the crack propagated very fast radially to the edges and with very low deflection less than ~ 0.3 mm. The radial cracks are demonstrated in Fig. S7 in SI. The membrane setup suggests that the high-porosity materials are more sensitive to the biaxial loading versus the pure bending tests with the beams. Wallboard is a highly porous material and is subject to many forces including bending. While this is not an exact representation of wallboard handling, the membrane test setup provides a simplified framework to test how porosity and multiaxial bending may influence damage susceptibility of wallboards. The stress concentration on void boundaries may be amplified due to the biaxial condition and thus lead to a lower stiffness when compared to the pure bending beams. Giovan et al.^23^ performed experiments on ceramics with uniaxial and biaxial bending and demonstrated that biaxial bending strength was ~ 8.1% lower than the one under uniaxial performance. They proposed that this was due to higher stresses near the flaws in tests with biaxial bending versus uniaxial. This is broadly consistent with the present membrane experiments, albeit here the strength reduction due to lower stiffness was much more pronounced. Gypsum is a very brittle material with a substantially lower Young’s modulus, by two orders of magnitude Dowling^20^, when compared to ceramics. Accordingly, high porosity (flaws) in the gypsum in the biaxial case may contribute to lower measured Young’s modulus in the membrane experiment.

### Load resistance of fiber-reinforced gypsum composites

The load resistance of gypsum composites reinforced with paper fibers, hemp fibers, and chopped fiberglass was evaluated over a fiber loading range of 0–2 wt%, with eight replicate specimens tested at each dosage. Fiber contents are reported as weight% relative to stucco.

Distinct reinforcement trends were observed among the three fiber types. Paper fibers and chopped fiberglass both exhibited a similar reaction under load, in which low fiber additions (0.5 wt%) resulted in strength values comparable to or below the gypsum control, while higher loadings produced measurable strength improvements. In contrast, hemp fibers did not provide reinforcement within the investigated range and instead led to progressive strength reductions as fiber content increased.

**Fig. 1:**
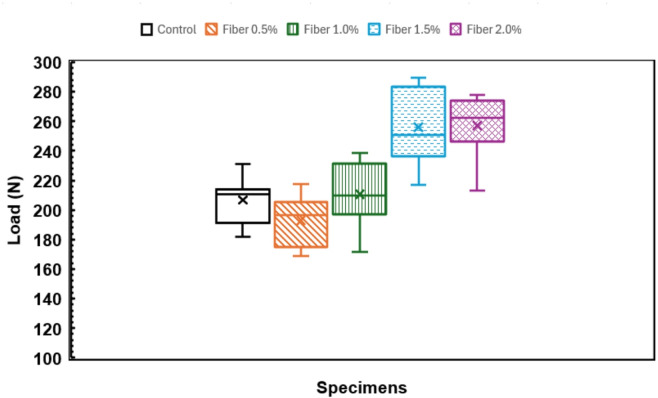
Maximum load sustained by the specimens with different loading of paper fibers.

**Fig. 2:**
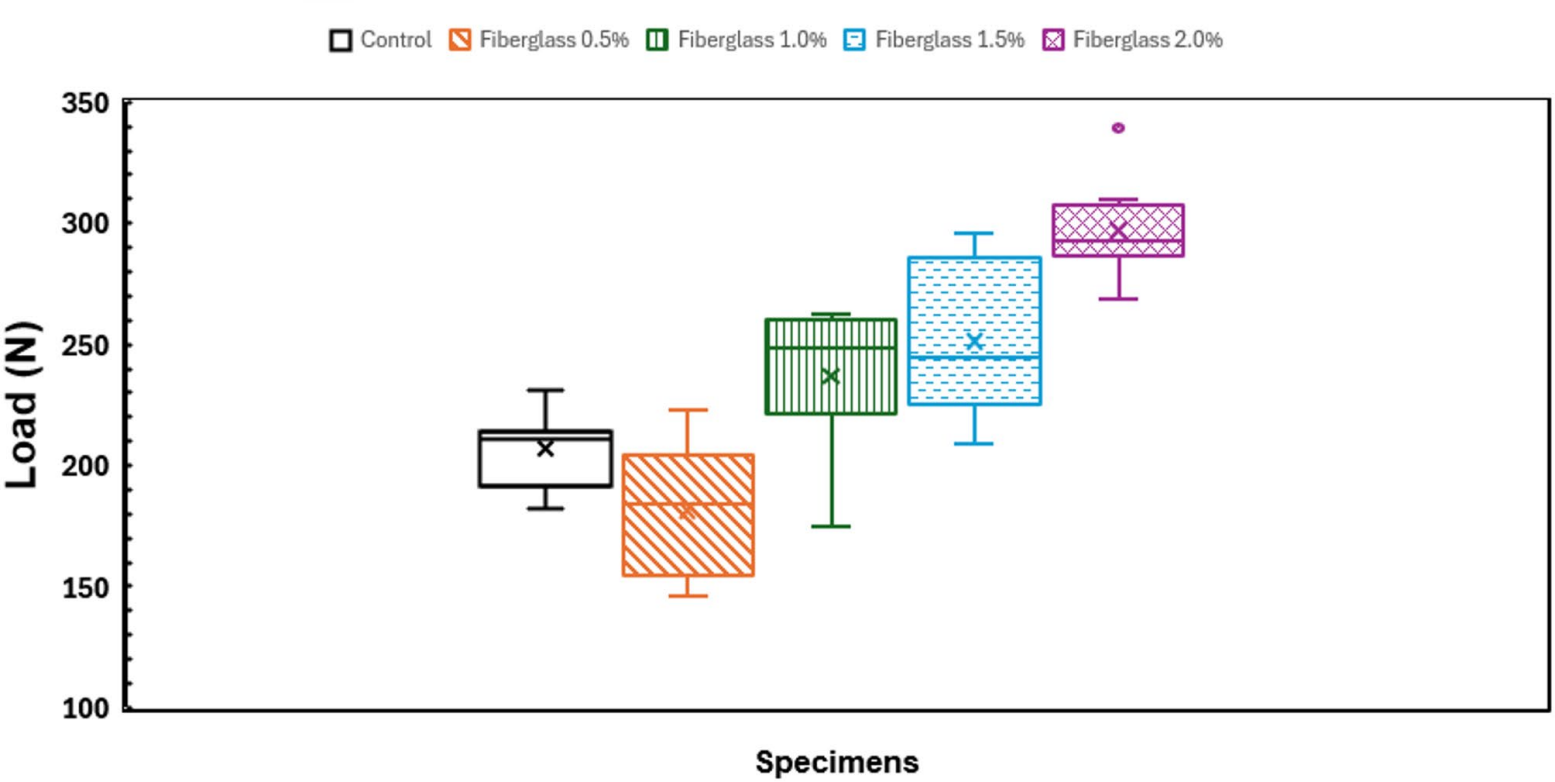
Maximum load sustained by the specimens with different loadings of chopped fiberglass.

Paper fiber reinforcement resulted in a maximum load increase of up to 24.2% at 2.0 wt% relative to the control as displayed in Fig. 1. However, a 0.5 wt% paper fiber addition reduced strength by 6.9%. indicating that a minimum fiber threshold may be required before effective stress transfer and crack-bridging mechanisms are activated. While the data presented does not directly resolve the underlying material mechanics, the trends observed are directionally consistent with the load transfer and crack bridging capabilities. Chopped fiberglass produced the largest strength enhancements, increasing maximum load by 14.2–43.2% as fiber loading increased as shown in Fig. 2. Similar to paper fibers, fiberglass also exhibited an initial strength reduction at 0.5 wt% (− 12.5%), consistent directionally with a threshold response for load-bearing improvement.

**Fig. 3:**
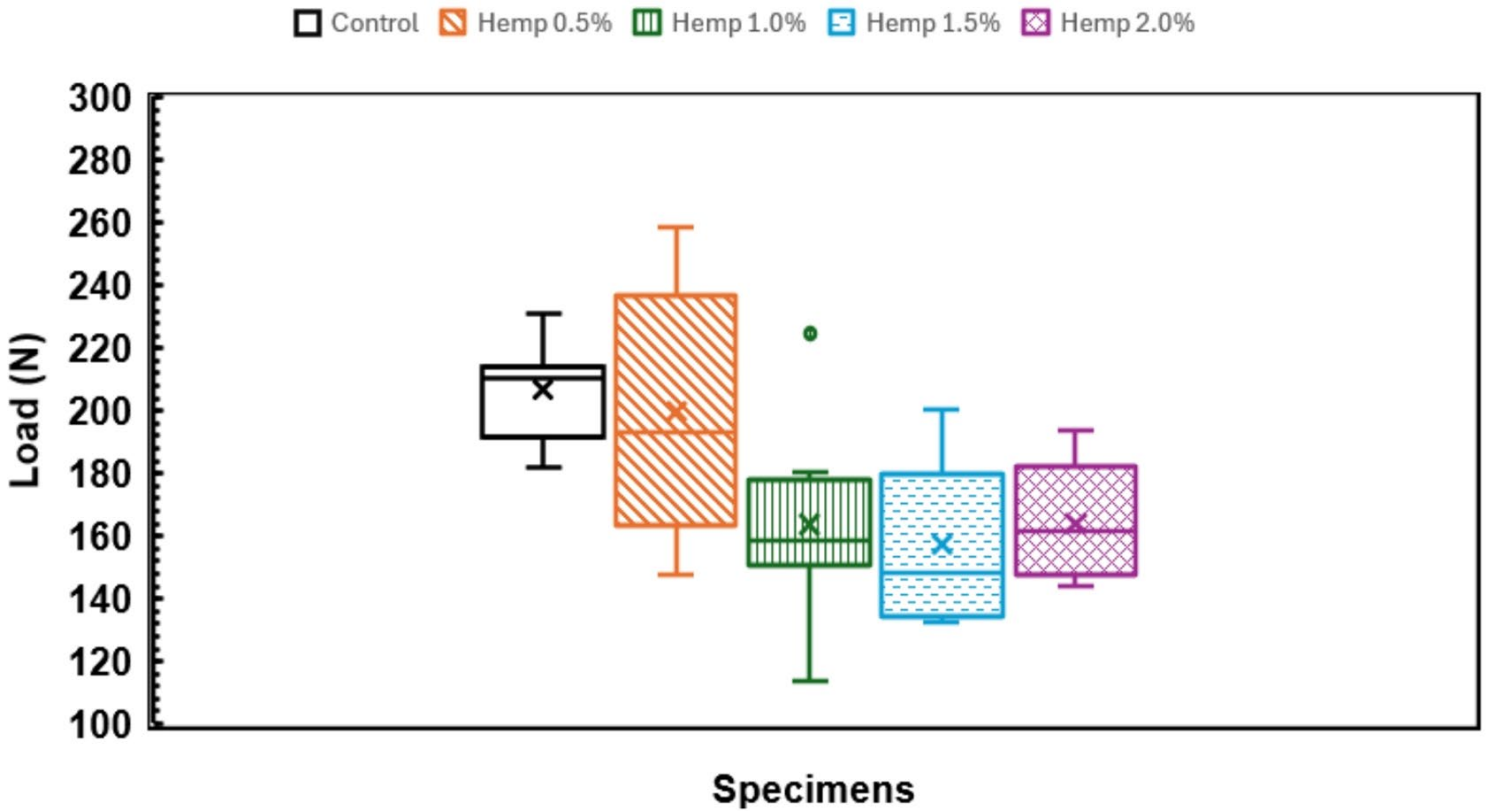
Maximum load sustained by the specimens with different loading of hemp fibers.

Hemp fibers consistently decreased load resistance by 3.6–20.8% over the investigated loading range as detailed in Fig. 3. The absence of strengthening may be related to the limited reinforcing efficiency of hemp fibers in the gypsum matrix, which is discussed further in the fiber morphology section.

Overall, the load resistance results imply that fiber length and reinforcement efficiency improve composite performance, with both paper fibers and fiberglass suggesting a minimum dosage is needed to outperform unreinforced gypsum. At low fiber contents (0.5 wt%), all fiber-reinforced specimens exhibited load resistance similar to the control, suggesting insufficient fiber participation in load transfer at these levels.

### Deflection of disks under central load

Deflection under central load was measured for disk membrane gypsum casts reinforced with paper fibers, hemp fibers, and chopped fiberglass to assess deformation capacity and post-peak behavior. Deflection provides insight into composite toughness and crack-bridging mechanisms beyond peak load resistance.

**Fig. 4:**
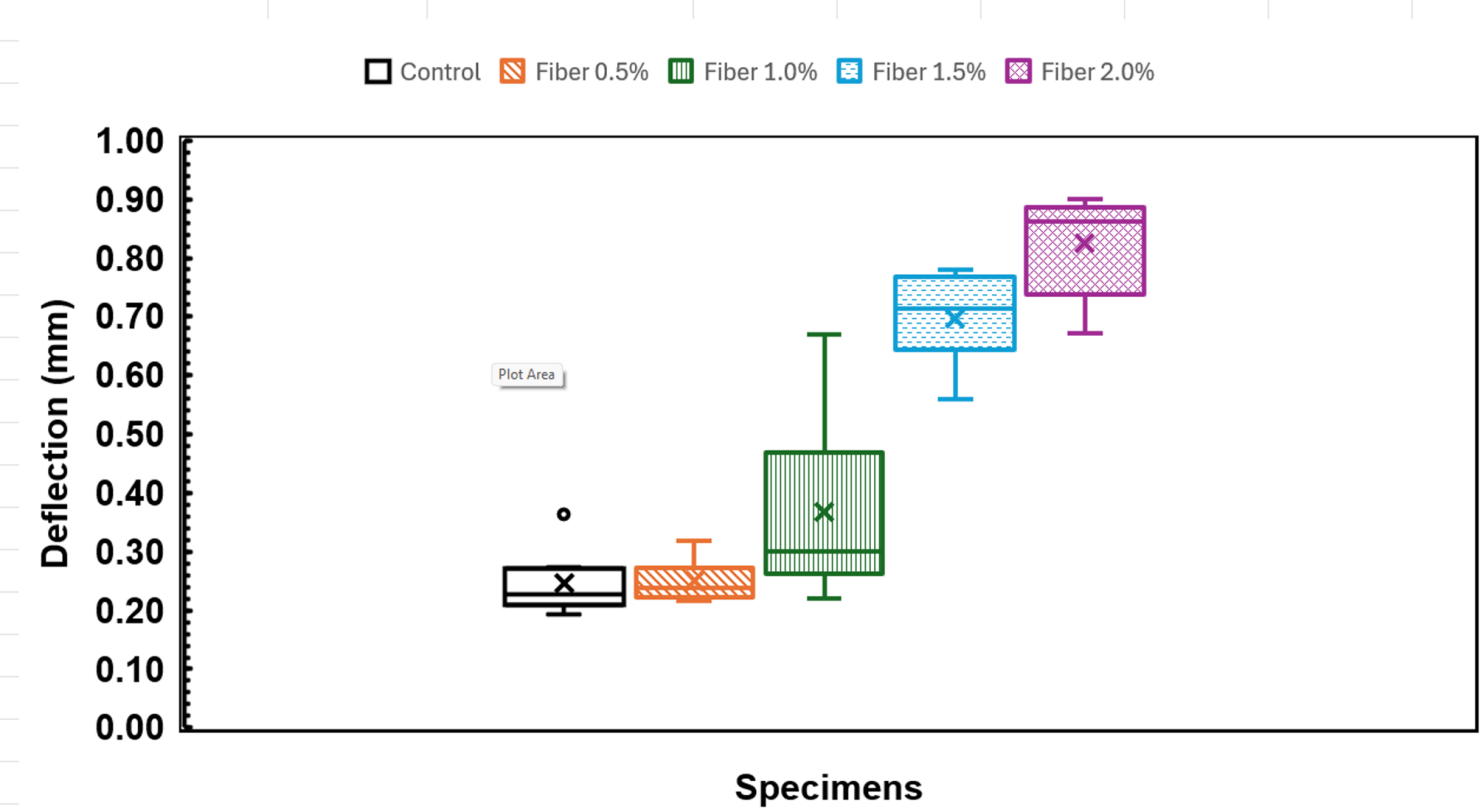
Deflection of disk-like casts reinforced with paper fibers at different loadings.

Paper fibers and chopped fiberglass both substantially increased deflection once a minimum fiber content was observed, whereas hemp fibers revealed limited and inconsistent improvements. For paper fiber–reinforced specimens, deflection increased by 1.4–234.7% relative to the control (Fig. 4), with higher fiber loadings allowing the disks to sustain greater deformation. The increased deflection results observed with paper fibers help maintain sample integrity even after the peak load was achieved. Contrary to the control where there is no load resistance after peak load, the paper fibers appear to allow the composite to carry load after gypsum matrix cracking.

**Fig. 5:**
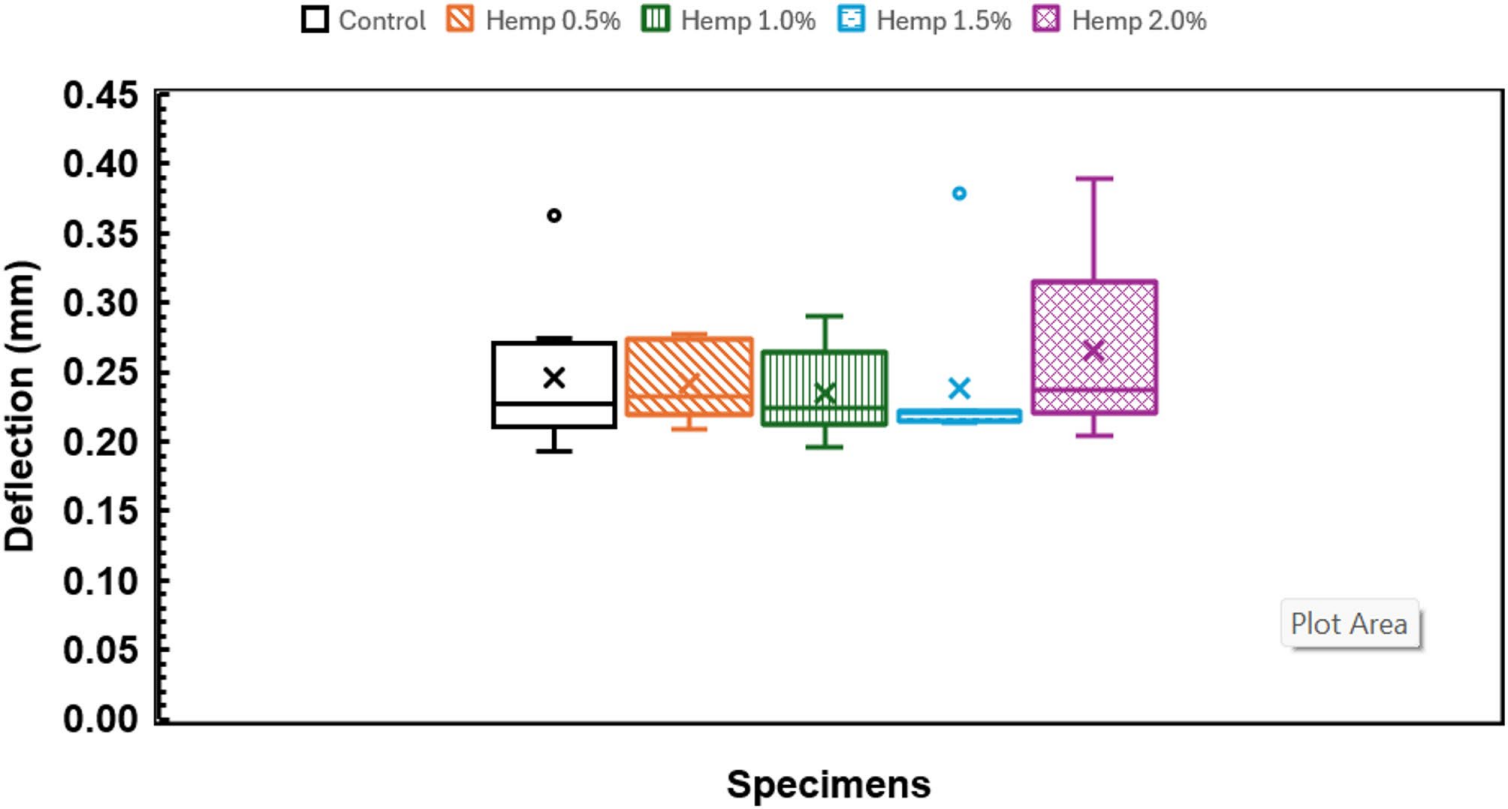
Deflection of disk-like casts reinforced with hemp fibers at different loadings.

Hemp fiber reinforced specimens exhibited deflection values that were generally lower than or comparable to the control, except at the highest fiber loading of 2.0 wt%, where a modest improvement of 7.6% was observed (Fig. 5). The large scatter in the deflection data further suggests inconsistent fiber engagement within the gypsum matrix, consistent with the absence of load resistance improvements.

**Fig. 6:**
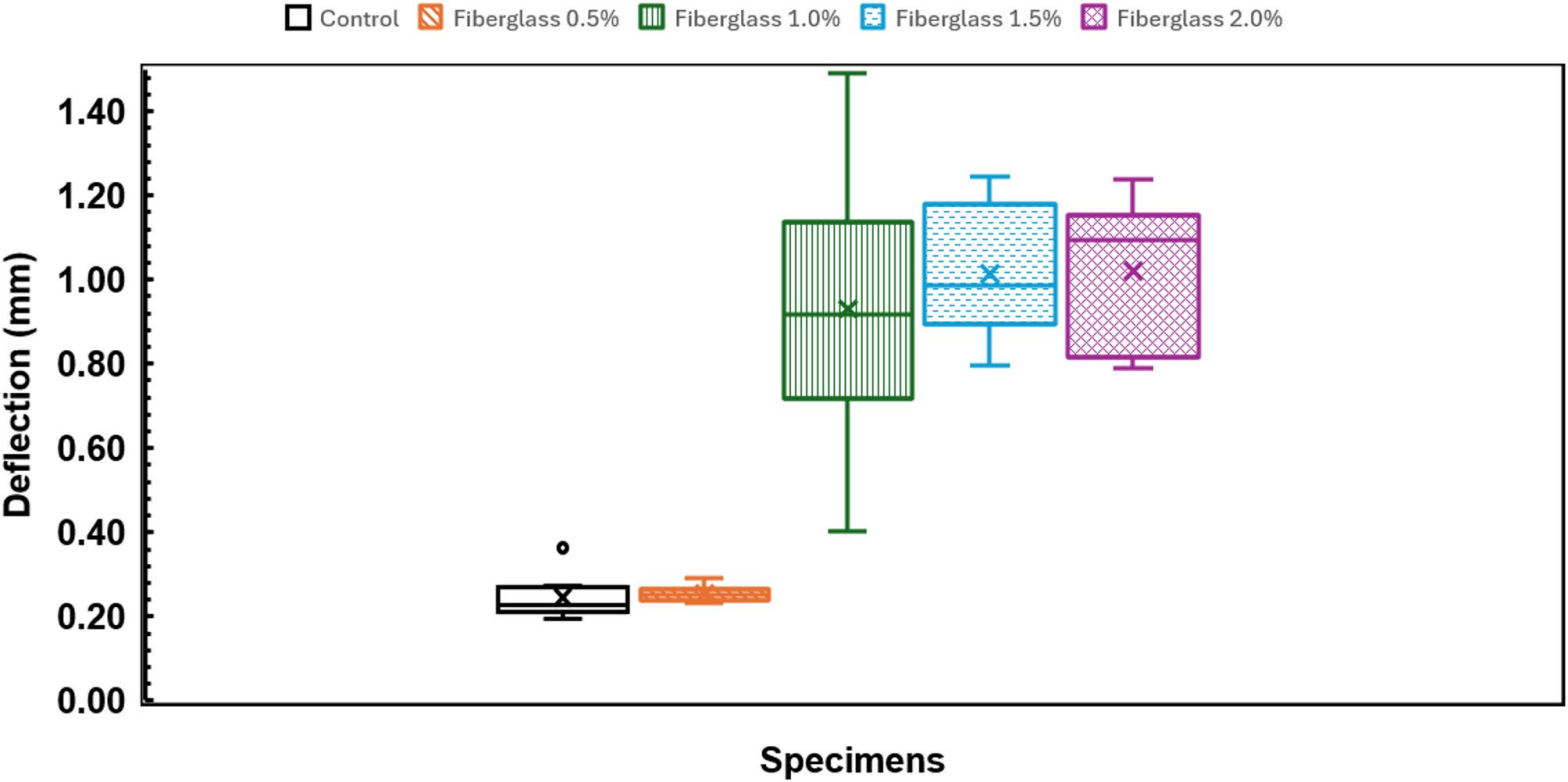
Deflection of disk-like casts reinforced with chopped fiberglass at different loadings.

Chopped fiberglass produced the most pronounced increase in deflection, with measured improvements ranging from 2.5 to 313.7% relative to the control (Fig. 6). At higher fiber loadings, fiberglass increased deflection by up to a factor of 3.1, reflecting even improved performance to carry force post peak load resistance as well as maintain the sample integrity.

Across all fiber types, deflection behavior at 0.5 wt% closely matched that of the gypsum control, aligning with the lack of load resistance improvement observed at low fiber contents. Load–deflection curves are consistent with a transition from gypsum matrix-dominated behavior to fiber-dominated behavior once the gypsum matrix fractures, as detailed in Fig. S8 left in SI. In specimens reinforced with paper fibers and fiberglass, the gypsum failed at similar loads to the control, after which fibers maintained specimen integrity and supported increased deflection and load. This transition was not clearly observed for hemp fibers.

When considered together, the load resistance and deflection results highlight fundamental differences between paper fibers and chopped fiberglass as reinforcing agents. Both fiber types exhibit a threshold dependent response, requiring sufficient fiber content before contributing to strength. Fiberglass provides the greatest enhancement in both load resistance and deflection, consistent with its longer fiber length. Paper fibers, while less effective than fiberglass, still significantly improve post-peak behavior and deformation capacity once a minimum fiber threshold is reached. In contrast, hemp fiber does not effectively reinforce the gypsum matrix within the studied loading range, which may suggest that fiber morphology and fiber length are important factors to improved load resistance. These results do not imply that hemp fibers cannot reinforce gypsum; rather the fibers utilized in this study at the tested range did not reveal strengthening.

### Fracture toughness of beams with 1.5% wt% fibers

Fracture testing is a function of specimen shape, the applied stress and the notch size, which acts as an initial stress concentrator to start crack propagation. Fracture toughness K is measured in the 3-point test of beam specimens, and the data processing method is described elsewhere in Dowling^20^.

**Fig. 7:**
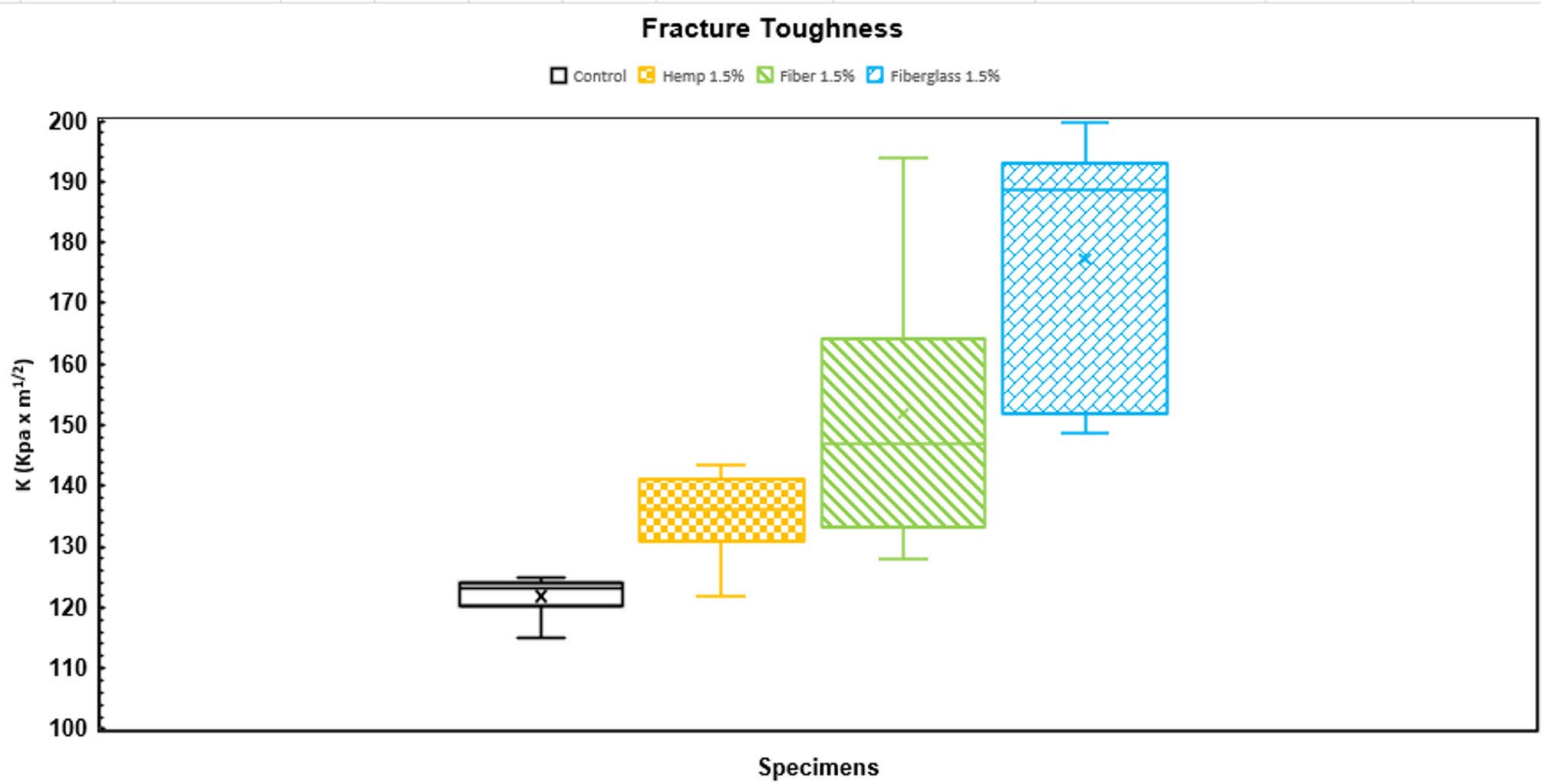
Fracture toughness of beams reinforced with hemp and paper fibers as well as chopped fiberglass.

The data on disk resistance to load obtained in the membrane test and described in the previous section suggests that the use of fibers is beneficial for a composite gypsum cast with paper fibers and fiberglass, rather than with hemp fibers used in this study. This trend is also revealed in the results of the fracture toughness tests with composite gypsum beams with 1.5 wt% fibers presented in Fig. 7. There were eight samples in each condition except only seven for paper fiber due to a broken sample. These results indicate that while hemp fibers modestly improved the fracture toughness by ~ 11%, paper fibers and fiberglass improved the fracture toughness more, by 25% and 45%, respectively, versus control. There is a higher range of variation in data with paper fiber and fiberglass, however, the trend of fracture toughness does improve versus both hemp and control.

## Fiber morphology

### Morphology of paper and hemp fibers

The results from the membrane tests with fiber-reinforced disk-like casts related to their loading and deflection presented in the proceeding section, revealed that paper and fiberglass fibers improved the mechanical properties of the surrounding gypsum matrix. However, hemp fibers did not reveal comparable reinforcement of the gypsum matrix. Accordingly, more analysis of hemp fibers in comparison with the other fibers is required to determine the reason for the poor performance of the former.

To determine fiber sizes, a MorFi tester was used. The MorFi tester is a common tool used in papermaking and analysis of fibers passing through a proprietary measurement cell. It is based on high resolution imaging at a known dilution and reveals the fiber lengths and diameters, as in Tourtollet et al.^24^.

**Fig. 8:**
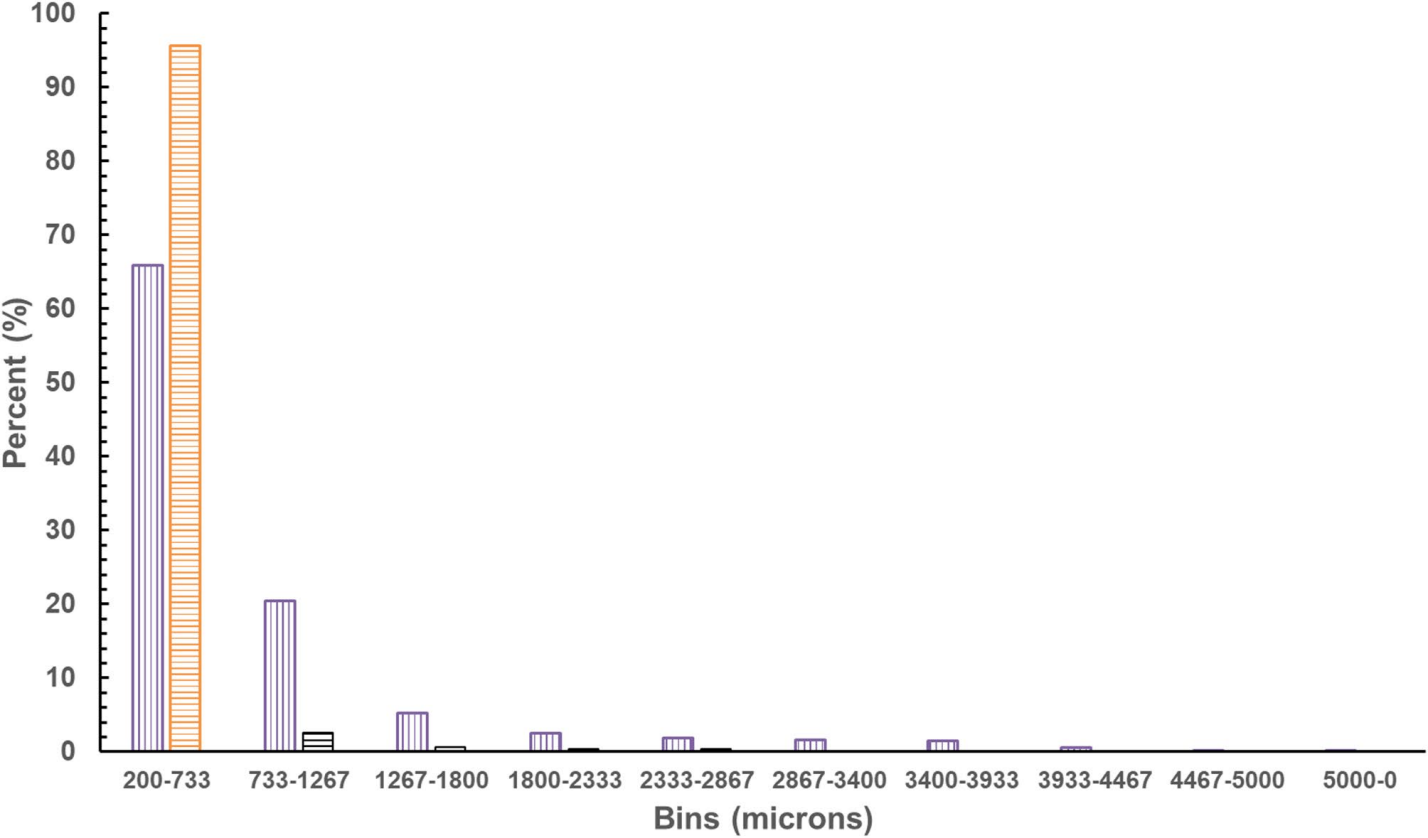
Fiber length-distribution obtained via MorFi analysis of paper and hemp fibers. MorFi segregates the fibers into bins within a range of fiber lengths. The first bin is between 200–733 μm in length as an example. Purple bars with vertical hatching correspond to paper fibers, whereas orange bars with horizonal hatching correspond to hemp fibers.

The analysis of the results in Fig. 8 reveals that the content of long hemp fibers is significantly narrower than that of paper fibers, practically over the entire fiber-length distribution. Accordingly, the ability of hemp fibers to reinforce the surrounding gypsum matrix is significantly diminished compared to that of paper fibers. The dependence of the reinforcement effect on the fiber length was also noted in Haba et al.^10^ in their analysis of the effect of palm fibers on gypsum matrix.

MorFi tester is set up to ignore fibers that have a width over 75 μm. The hemp fibers do appear coarser visually, and to better characterize them, another method of fiber observation was used. The Clark Classifier is a standard test used in paper making. The main function of such a tester is to classify fibers by their lengths which employ standard screen meshes in the testing apparatus. The screens used in the present tests were 14U, 30U, 50U, and 100U. A specimen of 5 g was weighed and passed through Clark Classifier. Hemp fibers were retained into bins, with the corresponding results summarized in Table 5.

**Table 5:** Hemp fibers characterized by Clark Classifier.

	Mesh size	Mesh opening (mm)	Specimen wt. + Filter paper (g)	Filter paper (g)	Specimen wt. (g)	Bin retain (%)
Bin 1	14U	1.41	3.04	0.79	2.25	44.9
Bin 2	30U	0.595	2.09	0.75	1.34	26.7
Bin 3	50U	0.297	1.41	0.78	0.63	12.6
Bin 4	100U	0.149	1.09	0.78	0.31	6.2
Mass balance loss (g)					0.48	9.6
Control weight (g)			6.12	1.11	5.01	

The results of MorFi testing revealed that there is a very low count of hemp fibers in comparison to paper fibers. However, almost 95.6% of hemp fibers were recorded in the first bin which represents fibers in the 0.200–0.733 mm range, which are short fibers. On the other hand, Clark Classifier revealed that almost 71.7% of hemp fibers by weight are long fibers greater than 0.595 mm and 44.9 wt% of the total are greater than 1.41 mm. These measurements suggest that a substantial fraction of hemp fibers is not classified as fibers and behaves like short, blocky particles than effective reinforcing fibers. To elucidate the notion of hemp fibers being non-functional filler, Scanning Electron Microscopy (SEM) was employed to observe their bonding within gypsum matrix.

### Morphology of gypsum-fiber matrices

Each SEM image was taken with both secondary electron (SE) and back-scattered electron (BSE) detectors. Two magnifications were selected to observe a single fiber loading level of 1.5 wt%. The 50$\times$ magnification provides a good resolution of the fiber distribution and a visual perspective on their count in gypsum matrix. The 2000$\times$ magnification reveals the gypsum needle interactions with fibers, as well as the overall fiber morphology.

**Fig. 9:**
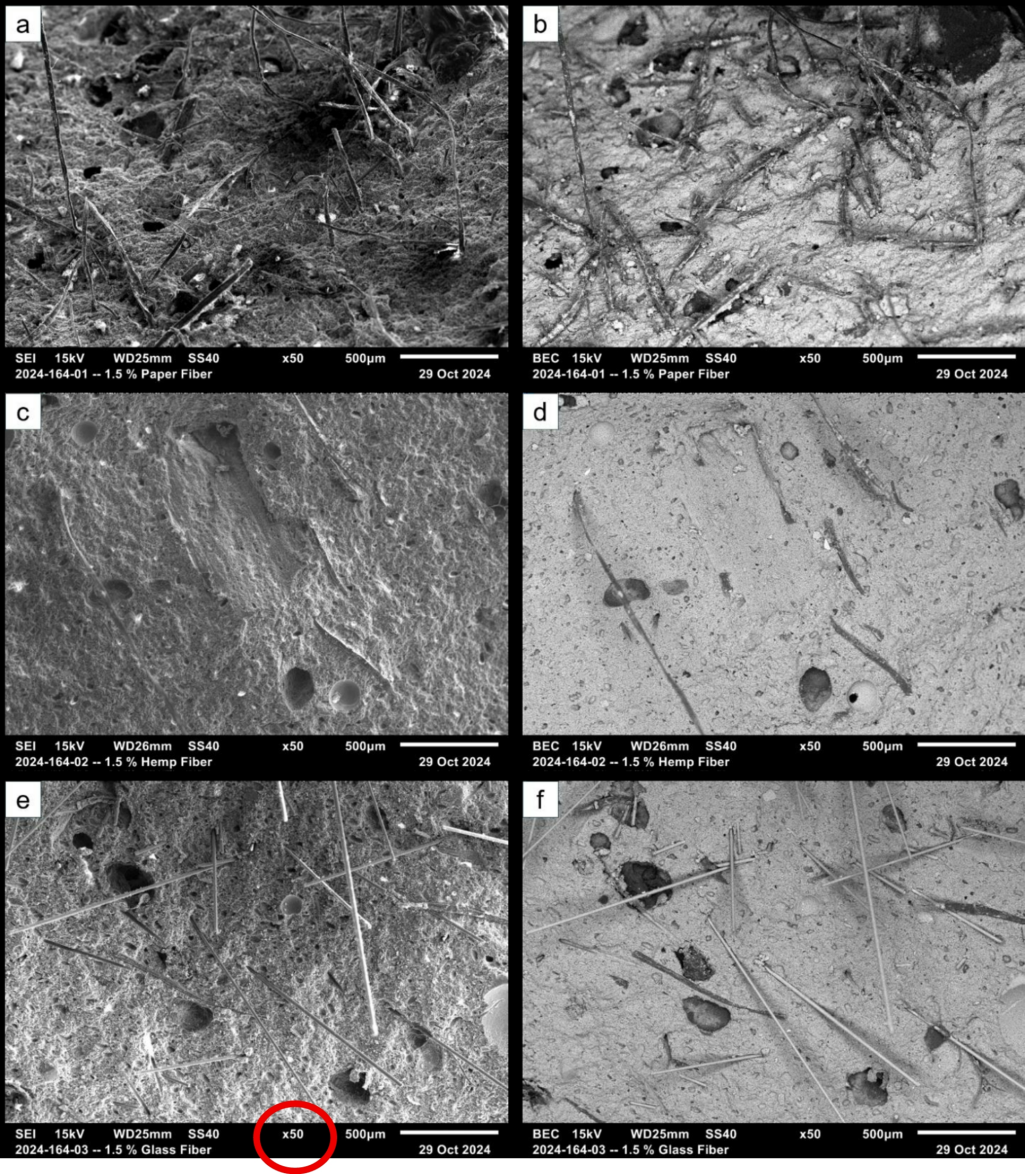
SEM images of fibers embedded in gypsum matrix at 50$\times$ magnification. Each specimen was carefully broken apart using chisel and a hammer, and a broken shard from the central portion of each specimen was taken for analysis. Prior to SEM analysis, each shard was glued to an aluminum stub and coated using carbon. SEM images of: (a)-(b) paper fibers, (c)-(d) hemp fibers, and (e) and (f) chopped fiberglass, all in gypsum matrix at 1.5 wt% loadings.

Figure 9, recorded under 50$\times$ magnification, illustrates that paper fibers appear ubiquitous when compared to hemp and fiberglass fibers despite being at the same loading levels of 1.5 wt%. Paper fibers appear strongly fibrillated and, consistent with MorFi testing, possess a high overall count and high surface area relative to hemp. It should be emphasized that the densities of paper, hemp and fiberglass fibers are approximately 1.0–1.1 g/cm^3^, 1.2–1.5 g/cm^3^ and 2.5–2.56 g/cm^3^, respectively, as documented by Jia et al.^25^. The volume of fiberglass fibers in gypsum matrix is by two orders of magnitude less than that of paper or hemp fibers.

**Fig. 10:**
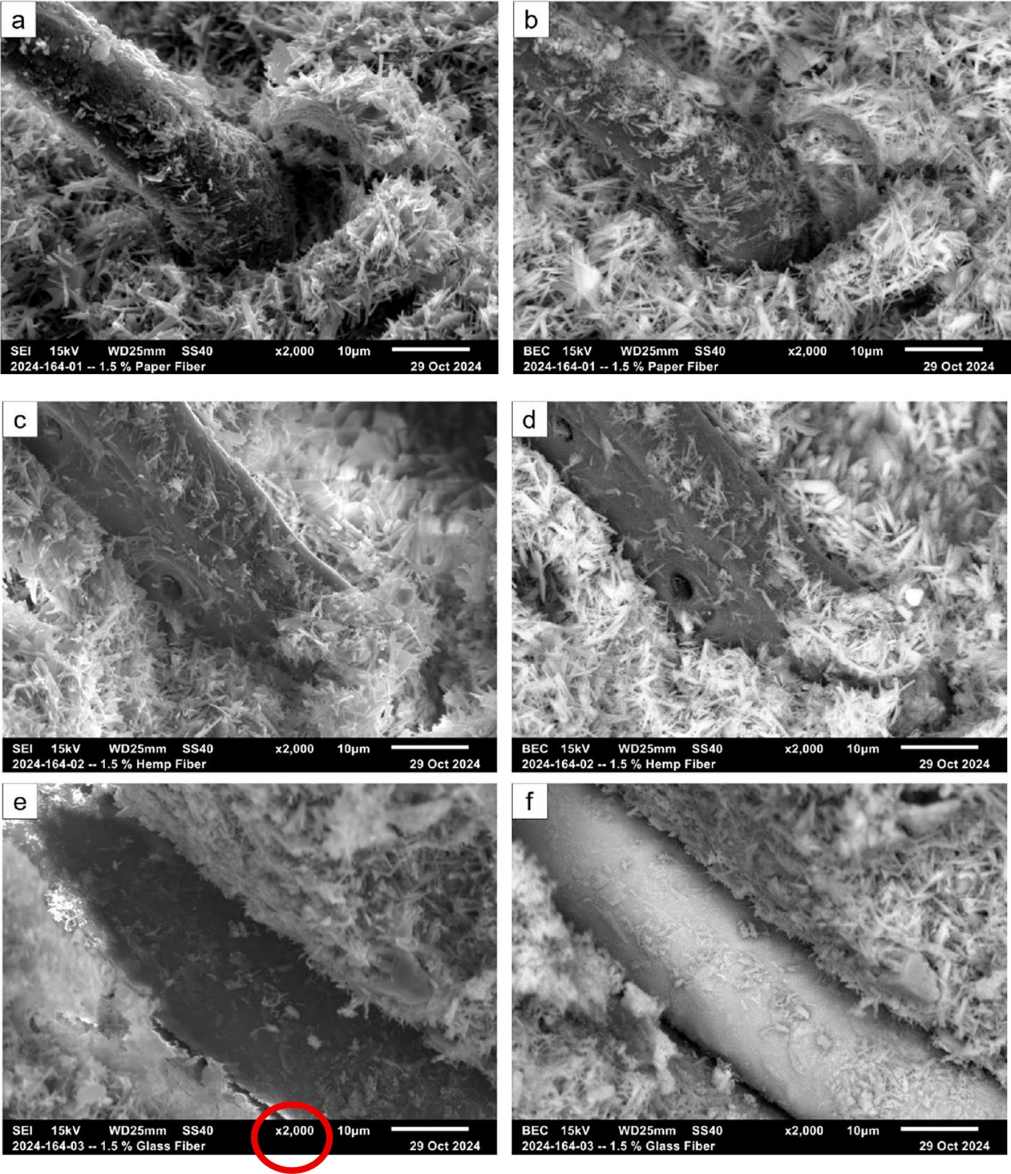
SEM images of fibers embedded in gypsum matrix at 2000$\times$ magnification. Each specimen was carefully broken apart using chisel and a hammer, and a broken shard from the central portion of each specimen was taken for analysis. Prior to SEM analysis, each shard was glued to an aluminum stub and coated using carbon. SEM images: of (a) and (b) paper fibers, (c) and (d) hemp fibers, and (e) and (f) fiberglass; all at 1.5 wt% loadings.

The examination of the same specimens at 2000$\times$ magnification in Fig. 10, indicates that paper fibers seem to facilitate gypsum crystals growth on them. To a lesser extent, hemp fibers also facilitate gypsum crystals growth. Accordingly, paper and hemp fibers appear to possess a much more compatible surface for gypsum crystal growth than fiberglass fibers.

Hemp fibers are visually blocky and do not appear to possess flexibility comparable to that of paper fibers. The short length and limited flexibility is likely contributors to the lower reinforcement of hemp fibers. Hemp fibers may require further refinement by chemicals as noted by Lucolano et al.^26^ and/or mechanical pulping to make a better reinforcing filler in gypsum matrices, although such modifications were not evaluated in this study.

Chopped fiberglass appears smooth and without gypsum crystal growth on the fibers. Fiberglass is known to involve sizing agents applied during manufacturing. Sizing is closely held formulation chemistries that fiberglass manufacturers apply on their products. As Thomason^27^ points out in his review paper, there are two chemistries that are typical for fiberglass manufacturing. One is the film forming additives used to help lubricate and hold fibers together during manufacturing. This lubrication also aids in promoting separation of the individual fibers when employed as a filler in a destination slurry or matrix. The other chemistry’s function is to promote coupling or bonding. This chemistry is almost always alkoxysilane which is used to promote bonding between the fibers and matrix to which they are blended into. These chemistries may have an impact on the overall interaction of fiberglass and the gypsum matrix. In Fig. 10e, one can observe a physical separation between fibers and gypsum matrix which suggests limited surface to gypsum bonding between gypsum and fiberglass.

If bonding is not the reason for improved composite flexibility at higher loads, then the length appears to be a plausible major contributor responsible for improved performance. The fiberglass fiber length is about 12.7 mm. MorFi tests revealed that only ~ 0.2 wt% of paper fibers are over 5 mm and 0 wt% of hemp fibers. This is an extreme physical difference between the three types of fibers explored here, and therefore is a good explanation for the measured improved performance, as also shown in Goncalves et al.^28^. Also, a study by Ali et al.^29^ demonstrated that fiberglass pulling out from a composite matrix is a function of the embedment fiber length. The glass fibers used by Ali et al. were 12.5 mm (1/2 inch) in length and thus, were fully embedded in gypsum. Ali’s experiments on E glass fiberglass revealed a maximum ultimate failure load of 19.8 N for a single fiber of 9.5 μm in diameter, while the embedment lengths were all less than 12.5 mm in their experiments. The loads were always less than 19.8 N signifying that fiberglass fibers were pulled out, rather than broken. The fiberglass in the present experiments possessed fibers of 16 μm in diameter, and during the loading and deflection tests, fiberglass revealed similar results consistent with fiber pull out rather breakage.

## Summary and conclusions

The load resistance and deflection responses of gypsum composites with three different types of fibers were studied in the present work. The results revealed the following findings:

1. Both paper fibers and fiberglass improve the load resistance and flexibility (deflection) properties of gypsum composites versus a control specimen without fibers. In this study, data suggests a threshold fiber content that is required for both types of fibers to reinforce the surrounding gypsum matrix. This threshold is between 0 and 1 wt%. This behavior is hypothesized to reflect the onset of effective crack propagation when the minimum fiber population is present in the gypsum matrix.
2. Hemp fibers require further refinement by chemical and/or mechanical pulping to make a better reinforcing fibers in gypsum matrices. Hemp fibers possess a much lower length than the other two types of fibers explored here. Even with refinement, hemp performance may remain below that of paper and fiberglass fibers due to the shorter fiber lengths, however this would require direct evaluation which was not conducted in this study.
3. Fiberglass fibers are 12.7 mm in length in this study, which is ~ 8 times that of the average length of paper fibers, and ~ 21 times that of hemp fibers. The length combined with the overall pull out resistance seems to improve performance of the composite material.
4. Fracture toughness was improved using all fibers; however, paper and fiberglass improved the toughness the most. A combination of the two types of fibers together will be the topic of future work to determine whether there is synergy to improve fracture toughness.
5. A biaxial bending membrane deflection test of pure gypsum and PMMA disks elucidated the importance of shear effects when cast gypsum is subject to biaxial bending by pointwise loads. This insight may be relevant to real world issues with damage occurring to wallboard when installing. Methods like fiber utilization to prevent damage may be a process improvement to facilitate wallboard durability.

## Supplementary Information

Below is the link to the electronic supplementary material.

Supplementary Material 1

Supplementary Material 2

## Data Availability

Detailed data used to plot the figures are available from the authors upon reasonable request.
