# Supplementary material for: Composite materials with recycled fibers: Evaluation of the effect of different fibers on gypsum composites
Source: Sci Rep. 2026 Apr 13;16:19010. doi: 10.1038/s41598-026-46588-6 (PMC13280370; doi:10.1038/s41598-026-46588-6)
Supplement: Supplementary file 2 — Supplementary Material 2 [file 41598_2026_46588_MOESM2_ESM.docx]

**Supplementary Information**

**Composite Materials with Recycled Fibers:**

**Evaluation of the Effect of Different Fibers on Gypsum Composites**

Mark Hemphill^1^ and Alexander L. Yarin^2^

^1^ United States Gypsum,

700 North US Highway 45 Libertyville, IL 60048-1296

^2^Department of Mechanical and Industrial Engineering,

University of Illinois at Chicago,

842 W. Taylor St., Chicago IL 60607-7022


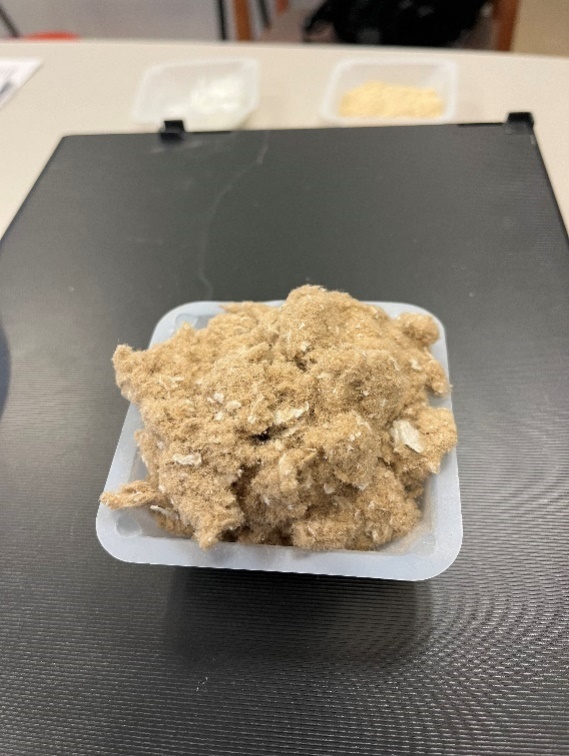

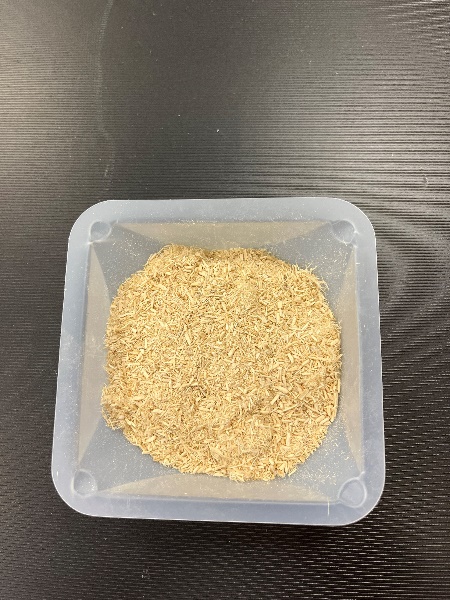

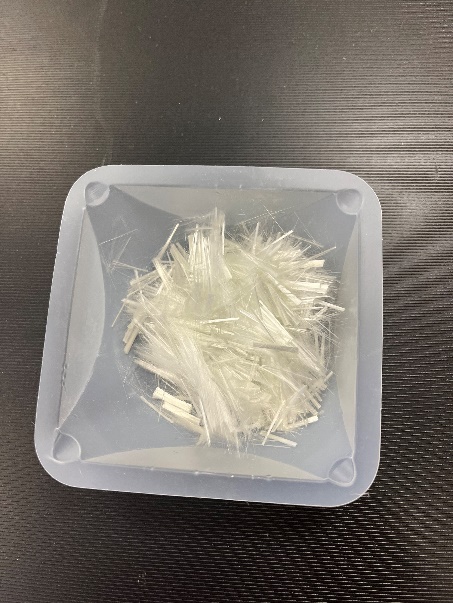


**Figure S1**. (Left) Six grams of paper fibers. (Center); Hemp fibers. (Right) Fiberglass.


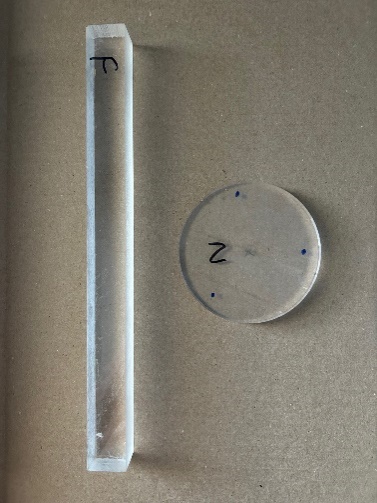


**Figure S2**: PMMA beam and disk

**Figure S3.** (Left) Waring blender; (Center) Disk forms; (Right) Fracture Toughness forms.


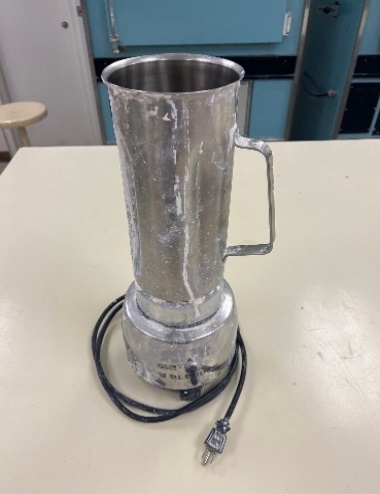

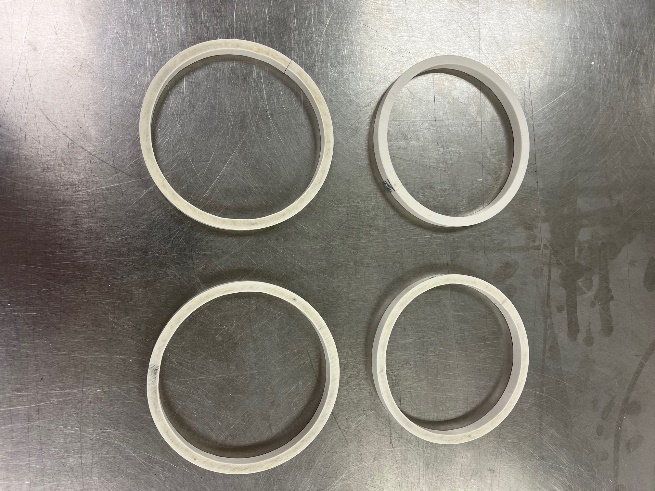

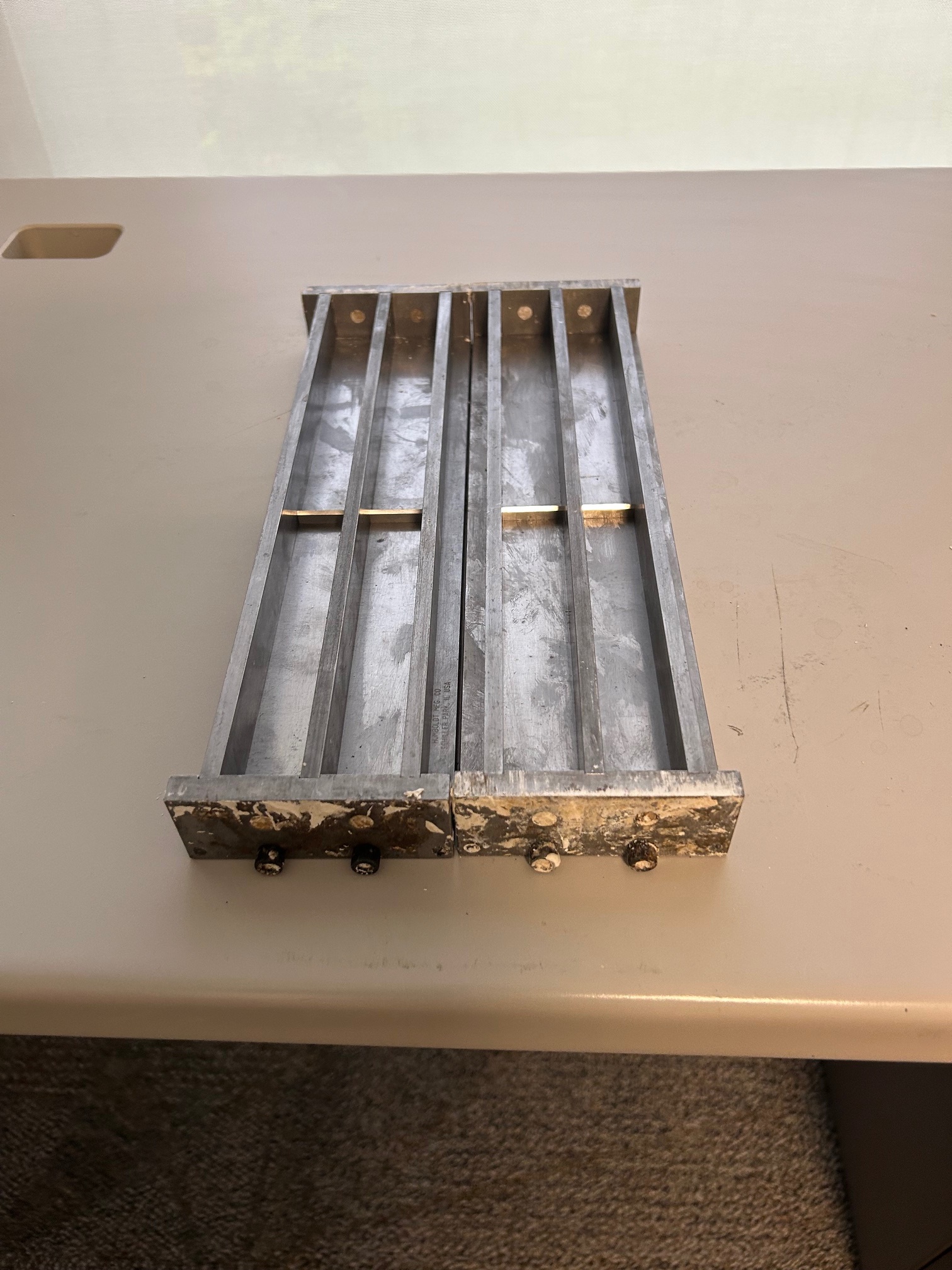


|  |  |  |  |  |
| --- | --- | --- | --- | --- |

**Figure S4.** (Left) Test setup with a disk in place; (Center) Support apparatus with a 3” hole; (Right) Fracture Toughness test with beam.


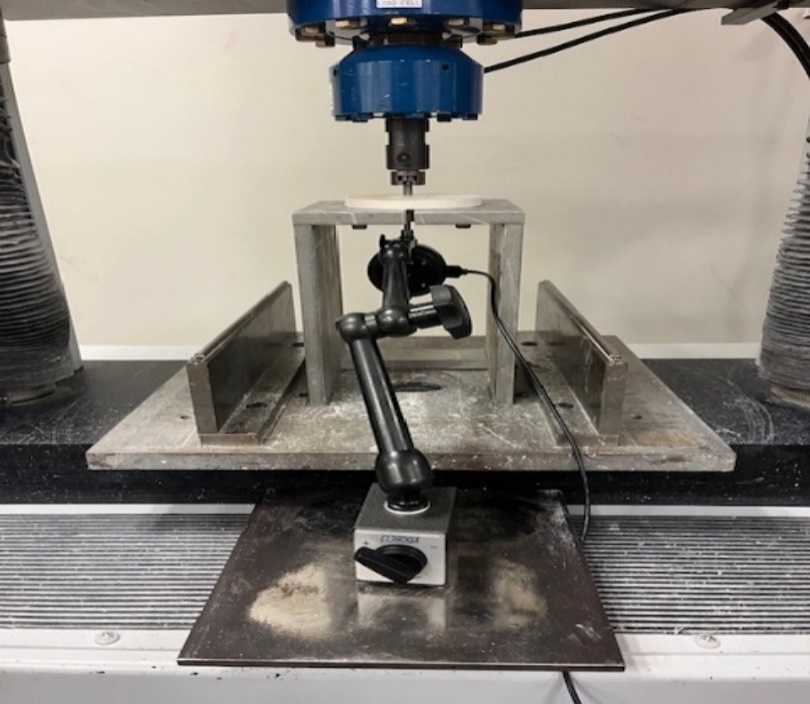

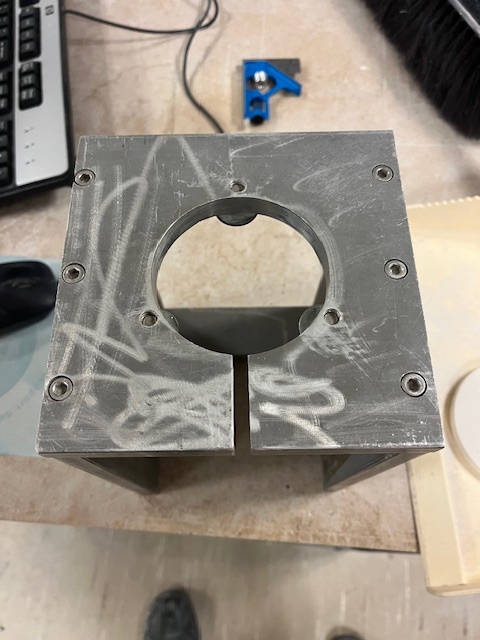

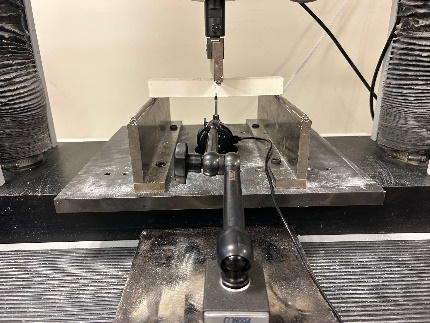


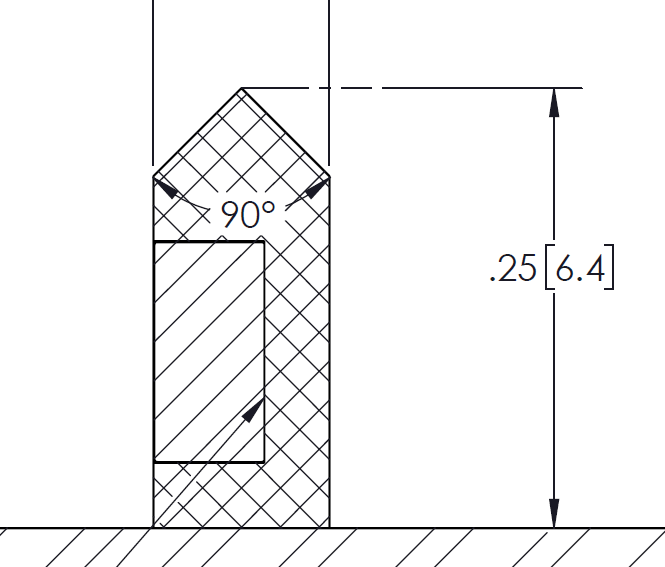

**Figure S5.** Notch detail 0.1” wide x 0.25” in height.


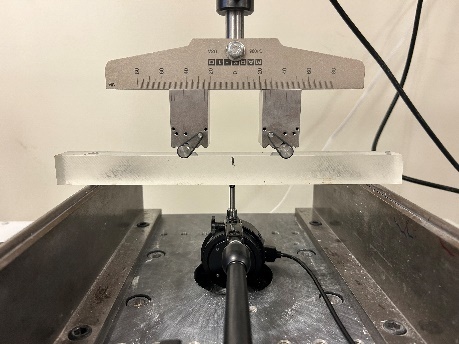

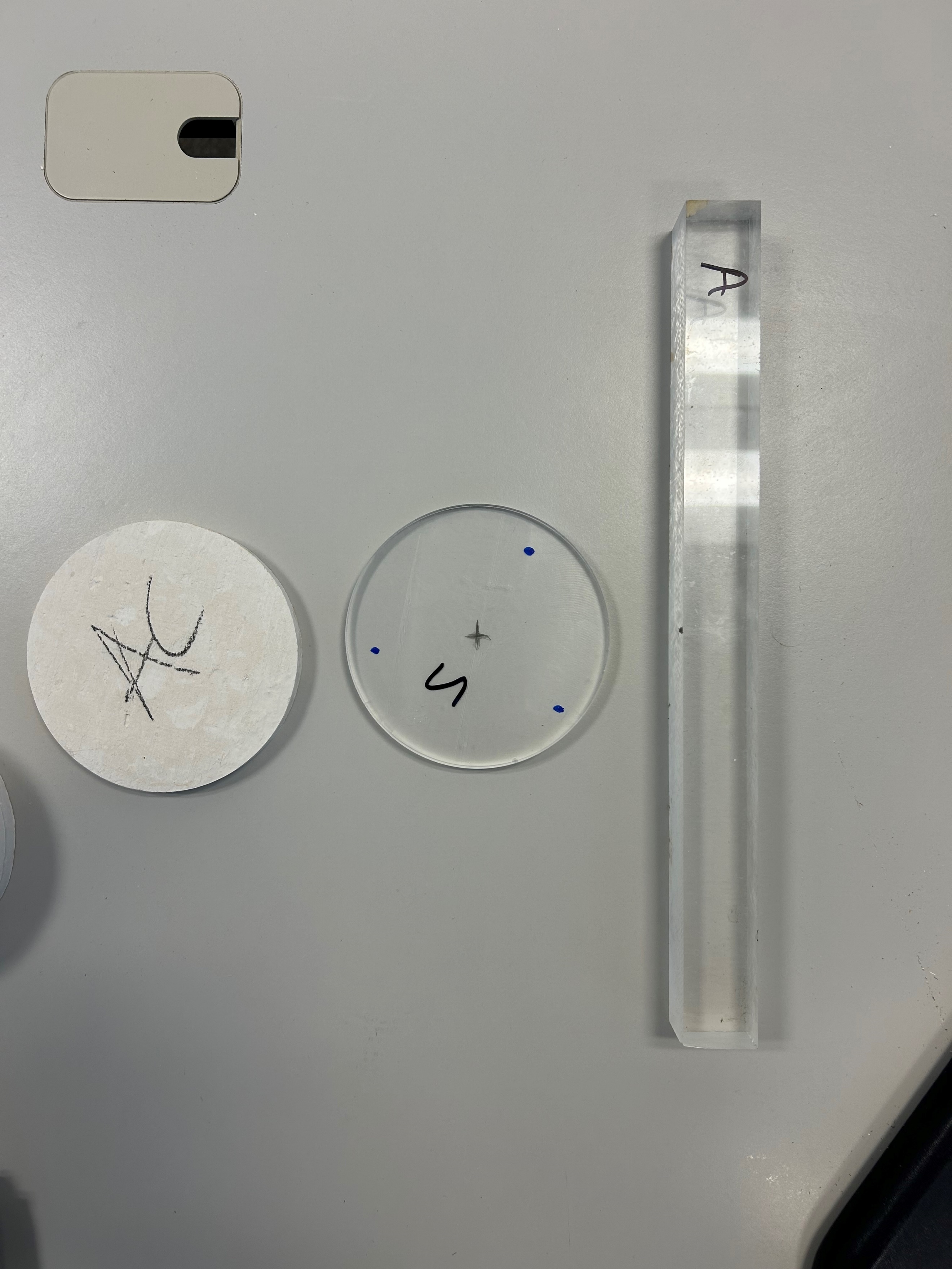


**Figure S6.** (Left) PMMA disk and beam beside a gypsum cast disk; (Right) PMMA beam in four-point bending test.


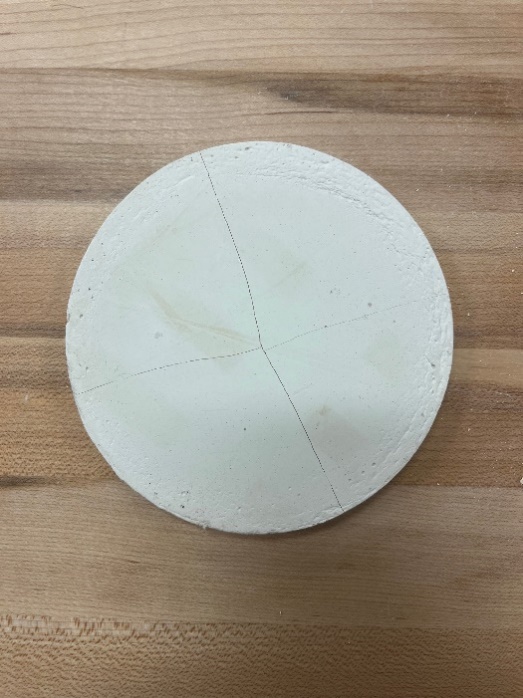

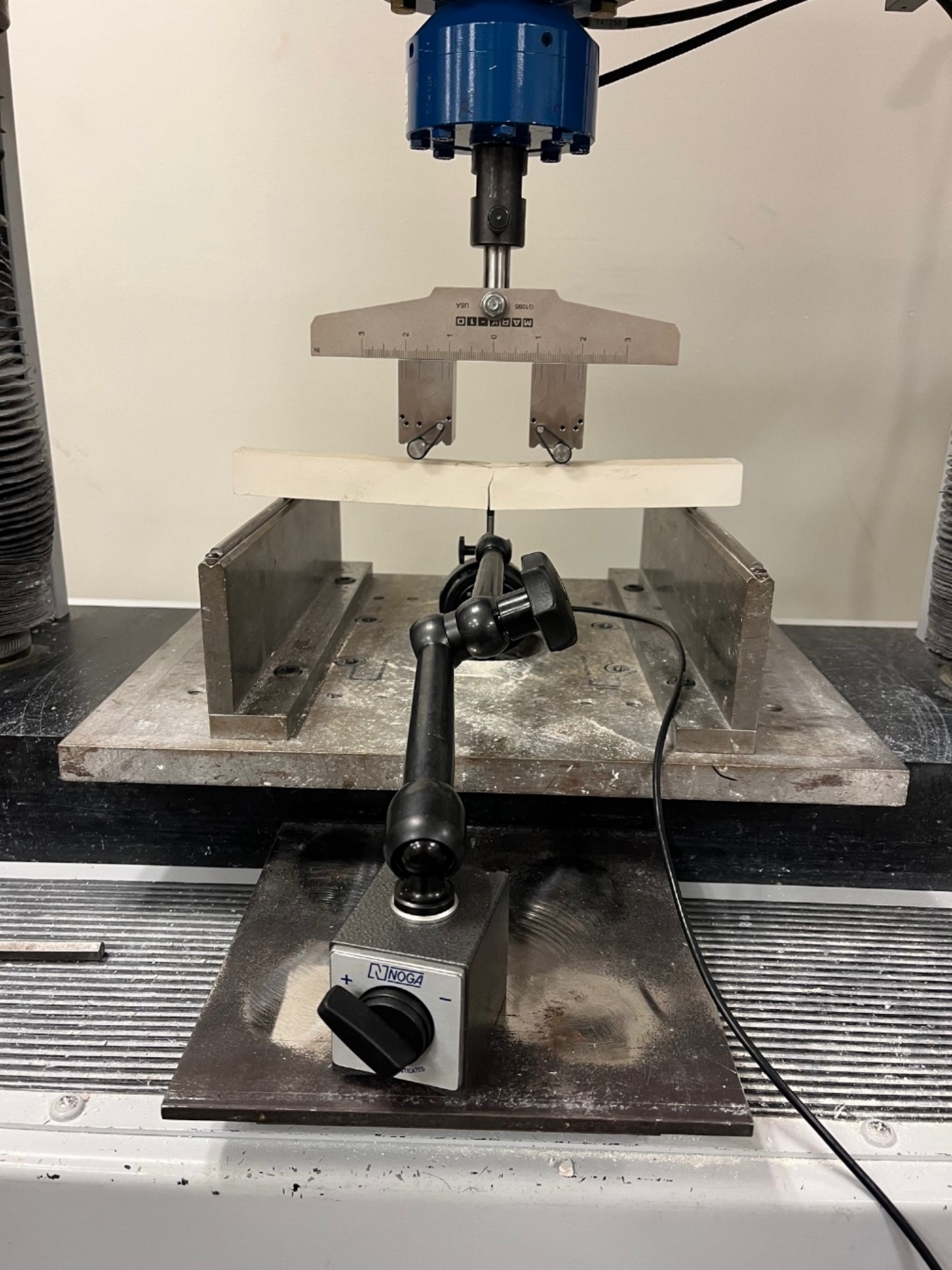


**Figure S7:** (Left) Gypsum disk revealing crack propagation from center radially outward (red arrow marking direction of crack propagation); (Right) Gypsum beam under 4 point bending (pure bending) revealing crack propagation from the stretching side to the loaded side.


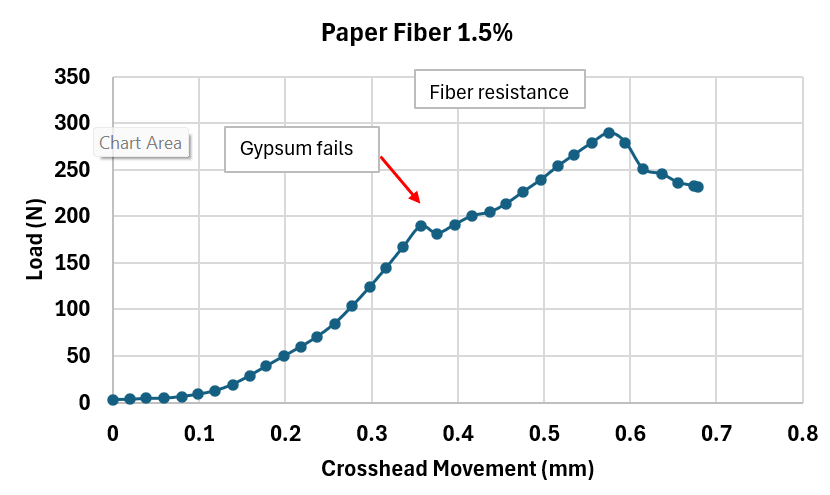

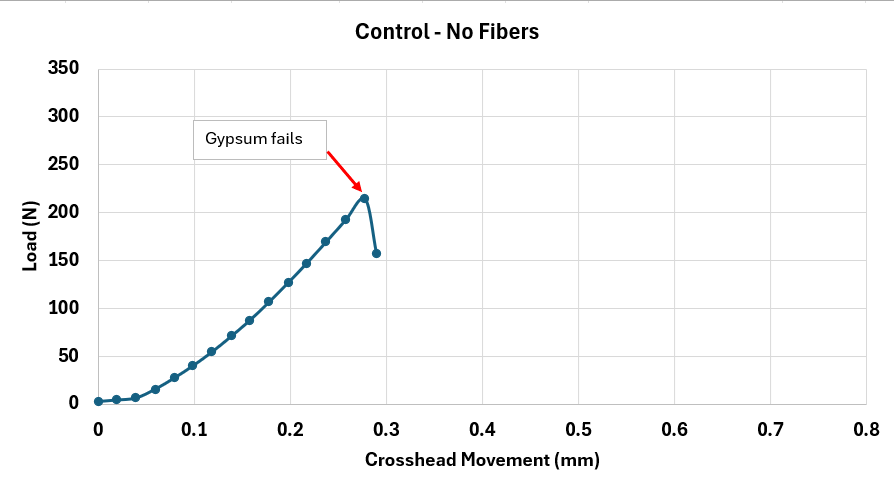


**Figure S8:** (Left) Gypsum control load/deflection dependence; (Right) Gypsum with 1.5% paper fiber load/deflection curve. After the initial ‘sagging’ part, both specimens reveal an approximately linear load/deflection dependence until gypsum fails, which implies a linear-elastic approximation of material behavior being plausible.
